# Lipidomic Analysis Reveals Differences in *Bacteroides* Species Driven Largely by Plasmalogens, Glycerophosphoinositols and Certain Sphingolipids

**DOI:** 10.3390/metabo13030360

**Published:** 2023-02-28

**Authors:** Eileen Ryan, Belén Gonzalez Pastor, Lee A. Gethings, David J. Clarke, Susan A. Joyce

**Affiliations:** 1 APC Microbiome Ireland, University College Cork, T12 K8AF Cork, Ireland; 2 School of Biochemistry & Cell Biology, University College Cork, T12 K8AF Cork, Ireland; 3 School of Microbiology, University College Cork, T12 K8AF Cork, Ireland; 4 Waters, Stamford Avenue, Altrincham Road, Wilmslow SK9 4AX, UK; 5 Division of Infection, Immunity and Respiratory Medicine, Faculty of Biology, Medicine and Health, Manchester Institute of Biotechnology, University of Manchester, Manchester M1 7DN, UK; 6 Faculty of Health and Medical Sciences, University of Surrey, Guildford, Surrey GU2 7XH, UK

**Keywords:** lipids, Bacteroides, pathways, plasmalogen

## Abstract

There has been increasing interest in bacterial lipids in recent years due, in part, to their emerging role as molecular signalling molecules. *Bacteroides thetaiotaomicron* is an important member of the mammalian gut microbiota that has been shown to produce sphingolipids (SP) that pass through the gut epithelial barrier to impact host SP metabolism and signal into host inflammation pathways. *B. thetaiotaomicron* also produces a novel family of N-acyl amines (called glycine lipids) that are potent ligands of host Toll-like receptor 2 (TLR2). Here, we specifically examine the lipid signatures of four species of gut-associated *Bacteroides*. In total we identify 170 different lipids, and we report that the range and diversity of *Bacteroides* lipids is species specific. Multivariate analysis reveals that the differences in the lipid signatures are largely driven by the presence and absence of plasmalogens, glycerophosphoinositols and certain SP. Moreover, we show that, in *B. thetaiotaomicron*, mutations altering either SP or glycine lipid biosynthesis result in significant changes in the levels of other lipids, suggesting the existence of a compensatory mechanisms required to maintain the functionality of the bacterial membrane.

## 1. Introduction

Bacterial lipids have recently emerged as influential contributors to the microbe–host molecular dialogue [1,2,3]. Lipids are hydrophobic or amphipathic small molecules found in all living cells, including bacteria, with important functions in membrane structure, energy storage and cell signalling [4]. Based on their chemical structures and biosynthetic origins, lipids have been grouped into eight categories; fatty acyls (FA), glycerolipids (GL), glycerophospholipids (GP), sphingolipids (SP), sterol lipids (ST), prenol lipids (PR), saccharolipids (SR), and polyketides (PK), and within each category, there are distinct classes and subclasses [5,6]. To date, over 40,000 biologically relevant lipids (mostly mammalian) have been listed to the LIPID MAPS Structural Database [7]. However, there is growing interest in cataloguing non-mammalian lipids, including those produced by gut microbes, prompted, in part, by the identification of some bacterial lipids as molecular signals [1,3,8]. 

*Bacteroides* are early colonisers of the mammalian gut, establishing stable, long-term, and generally beneficial interactions with their human host [9]. *Bacteroides* have been shown to produce a variety of important bioactive lipids, including sphingolipids (SP) and N-acyl amines called glycine lipids [10,11,12,13,14,15,16,17,18,19]. It is now well recognised that *Bacteroides* are one of only a few bacterial genera that produce SP [13,20,21]. *B. fragilis* generates a bioactive SP (α-galactosylceramide, α-GalCer), which binds to the antigen-presenting protein, CD1d, thus influencing the number and function of natural killer T cells (NKT-cells) in the intestine, with consequences for the progression of colitis in a murine model [10,11]. In another study, the lack of bacterial SP production was shown to promote intestinal inflammation, along with concurrent changes in the host SP pool [13]. Indeed, SP produced by *Bacteroides* have been shown to cross the epithelial barrier and impact hepatic SP pools [21]. 

Glycine lipids (GlyL) are a family of lipids derived from the initial *N*-acylation of glycine, which results in the production of a mono-acylated glycine molecule called commendamide [16,22]. Commendamide is further modified by an *O*-acylation, resulting in a diacylated glycine with additional modifications, to generate a family of glycine lipids that includes a serine-glycine dipeptido-lipid (flavolipin, FL) and a large complex glycine lipid called Lipid 1256 [18,23,24,25]. Interestingly, commendamide was originally identified in a screen for agonists of GPCR G2A/132 that result in increased levels of NF-kB expression [16]. GlyL, FL and Lipid 1256 have been reported to signal to eukaryotic cells by engaging TLR2, promoting the production of pro-inflammatory cytokines [14,15,17,18,19]. 

It is clear, therefore, that *Bacteroides* may produce unique lipids with the potential to signal to the mammalian host. However, a comprehensive examination of the *Bacteroides* lipid signature has not been conducted, and this limits a full appreciation of their potential in the host–microbe dialogue. In the present study we describe the pathways and compare the lipid signatures of four important species of *Bacteroides*: namely *B. thetaiotaomicron*, *B. fragilis, B. ovatus* and B. *vulgatus* (recently elevated to *Phocaeicola vulgatus*), with a particular focus on the bioactive SP and glycine lipids. We identify 170 different lipids and show that the lipid signatures vary in a species-dependent manner. In addition, we show that mutations in SP or glycine lipid biosynthesis significantly change the lipid signature of *B. thetaiotaomicron*, and these compensatory changes need to be considered when studying the role of these lipids in *Bacteroides* and in the microbe-host dialogue.

We describe, for the first time, a comprehensive and qualitative comparison of the lipid signatures of four important *Bacteroides* species. We identify a group of *Bacteroides* core lipids and uncover species-specific differences in plasmalogen, glycerophospholipid and sphingolipid metabolism, with more subtle differences observed in glycine lipid production. This data will provide a useful platform for the further characterisation of the lipid-based host–microbe dialogue and the influence of microbial lipids on host health and disease states. 

## 2. Materials and Methods

### 2.1. Materials

Organic solvents (Supelco 2-propanol and acetonitrile) used for the extractions or precipitations and mobile phase preparation were hypergrade for LC-MS LiChroslv^®^ and obtained from Merck (Darmstadt, Germany). Buffers used for mobile phase preparation were from Fisher Chemical Optima™, LC-MS grade Formic Acid from Fisher (Leicestershire, UK) and LC-MS grade LiChropur™ ammonium formate from Merck (Darmstadt, Germany). Internal standard, applied for normalization to each sample, N-palmitoyl-D-erythro-sphingosylphosphorylcholine (16:0 SM, Avanti Polar Lipids, powder) was purchased from Merck (Darmstadt, Germany).

### 2.2. Bacterial Strains and Growth Conditions 

*B. thetaiotaomicron* VPI-5482 ∆*tdk* (gift from Eric Martens), *B. fragilis* 638R (gift from Eduardo Rocha), *B. ovatus* ATCC8483 ∆*tdk* (gift from Eric Martens and Nicole Koropatkin) and *B. vulgatus* ATCC8482 (gift from Eric Martens) were anaerobically cultured at 37 °C in brain heart infusion (BHI) medium (Sigma) supplemented with hemin (5 μg mL^−1^), 0.1% (*w/v*) cysteine and 0.2% (*w/v*) sodium bicarbonate. The *B. thetaiotaomicron* ∆*SPT* mutant was a gift from Eric Brown [13], whilst the *B. thetaiotaomicron* ∆*glsB* mutant, disrupted for N acyl transferase, was constructed as previously described [25]. 

### 2.3. Lipid Extraction

Bacteria were inoculated in BHIS broth, and after 24 h, cultures were normalised to 1OD_600_, then pelleted by centrifugation at 8000 rpm × 10 min. Pellets were washed twice with phosphate bufferered saline (1xPBS). Bacterial pellets were subject to a single phase isopropanol lipid extraction, and the protein percipitation procedure was as described by Sarafian et al. [26]. This methodology represents a one-step, one phasic extraction method that is shown to precipitate proteins and to extract a broad range of lipids of different polarity [27]. Briefly, washed pellets were resuspended in isopropanol (to a final density of 1OD_600_ mL^−1^) with added internal standard (16:0 SM, 1 µg/mL) for normalization purposes. Samples underwent vortexing for 30 s, then they were incubated at room temperature for 10 min, with occassional mixing, before overnight storage at −20 °C. The supernatent was collected following centrifugation for 20 min at 14,000× *g* and stored at −80 °C for LC-MS analysis.

### 2.4. LC-MS Conditions

Bacterial isopropanol lipid extracts were analysed using Waters Xevo™ G2-XS QTOF Mass Spectrometer coupled to a Waters ACQUITY^TM^ UPLC^TM^ system. Extracted samples were injected (5 μL injection volume) onto an ACQUITY CSH™ column (100 mm × 2.1 mm, 1.7 μm; Waters) at 55 °C with a flow rate of 400 μL/min. The mobile phases consisted of phase A (acetonitrile/water (60:40, v:v) with 10 mM ammonium formate and 0.1% formic acid), which was subject to gradient mixing with mobile phase B (isopropanol/acetonitrile (90:10, v:v) containing 10 mM ammonium formate and 0.1% formic acid) (Table S1 in the Supporting Information details the gradient parameters). 

Mass spectrometry was performed under both positive and negative ESI modes using the following paramaters: Acquistion mode: MS^E^; acquistion range: from *m*/*z* 100 to 2000; acquisition time: 1 s/scan; source temperature: 120 °C; desolvation temperature: 550 °C; nitrogen gas flow: 900 L/h; capillary voltage: 2.0 kV (positive mode) or 1.5 kV (negative mode); cone voltage: 30 V. For both ionization modes, leucine enkephalin (*m*/*z* 556.2771 in ESI+, *m*/*z* 554.2615 in ESI−) was continuously infused at 30 μL/min and sampled every 30 s for lock mass correction.

### 2.5. Characterisation of Plasmalogens

Acid hydrolysis was applied to confirm the presence of plasmalogens in *B. thetaiotamicron* extracts according to Murphy et al. [27]. Briefly, isopropanol was removed from lipid extracts under nitrogen, and eppendorfs were inverted over five drops of concentrated HCl in a test tube cap for 5 min. This caused the complete hydrolysis of the vinyl ether bond of the plasmalogens, while the diacyl ester bonds remained intact, a confirmatory feature of plasmalogens. The samples were re-extracted with isopropanol then reanalysed by LC-MS, as described above. 

### 2.6. MS Data Processing and Statistical Analyses

For targeted analysis, MS data was processed using the open-source, Skyline-daily (beta) freeware (MacCoss Lab Software, University of Washington, Washington, DC, USA). An in-house *Bacteroides* lipid library was constructed from mining the existing literature, to include classes of sphingolipids, *N*-acyl amines, fatty acids, and glycerophospholipids with cross checking of raw data in both ionisation modes for accurate mass (where delta mass < 5 ppm), retention time, and MS/MS fragmentation, where possible. This database, together with LC-MS raw data files, was uploaded into Skyline for processing following lock mass correction. Both positive and negative mode data were processed, following application of a correction factor (based on peak intensity of several common lipids detected in both modes), then normalised to the internal standard. Data was log-transformed using MetaboAnalyst 5.0 web-based platform [28,29], prior to multivariate (principle component analysis (PCA), heatplot representation, ANOVA statistical scrutiny) and univariate (volcano plots) analysis. Customised python scripts were applied to produce volcano plots. GraphPad Prism 5.0 was used to produce bar charts and for regression analysis. For untargeted analysis, raw LC-MS data files were first processed (peak alignment) using Progenesis QI (Waters^TM^, UK) for both positive mode and negative mode data and then imported into MetaboAnalyst 5.0 for median normalisation, log transformation and multivariate analyses.

## 3. Results

### 3.1. The Lipid Signatures of Bacteroides Are Species Specific

**Figure 1:**
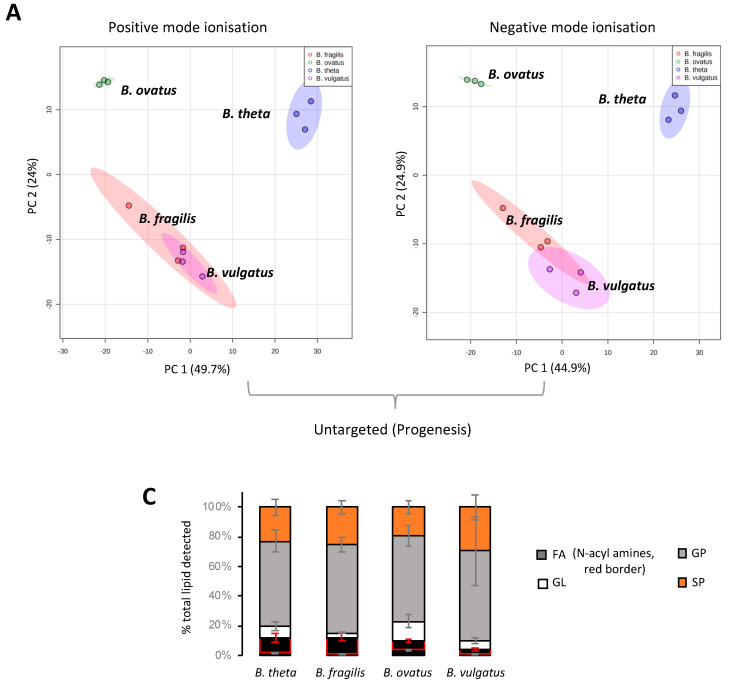

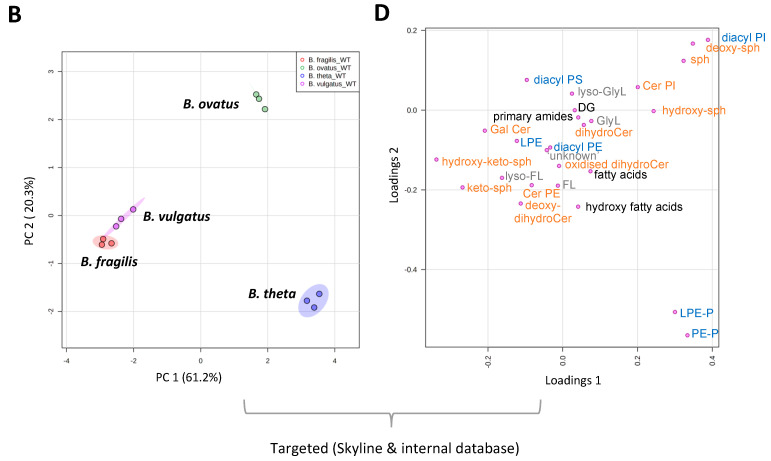
**LC-MS and multivariate analysis suggest that *B. fragilis* and *B. vulgatus* may have similar lipidomes, which are distinct from both *B. thetaiotaomicron* and *B. ovatus*.** (**A**) PCA score plot generated from untargeted lipid analysis from both positive or negative mode ionisation data; LC-MS raw data was imported into Progenesis QI, data were median-normalised and log-transformed using MetaboAnalyst 5.0, a web-based platform, prior to multivariate analysis. (**B**) PCA score plot generated from targeted lipid analysis; LC-MS raw data was uploaded into Skyline and processed against our internal lipid database for both positive and negative ionization modes. A correction factor was applied to negative ionization mode data, to combine it with positive ionization mode data, before normalization to the internal standard. Data were then log-transformed using MetaboAnalyst 5.0 wed-based platform prior to multivariate analysis. PC1 represents the maximum variance direction in the data followed by PC2. PC1 and PC2 allow relative distance visualization of each dataset to each other. (**C**) Relative distribution of fatty acyls (FA), glycerolipids (GL), glycerophosholipids (GP) and sphingolipids (SP) among the four *Bacteroides* species. Data are represented as the mean ± standard deviation (SD) of three independent biological experiments (**D**) The equivalent Loadings plot of (B) reveals differences in the lipid profile of *Bacteroides* driven largely by plasmalogens (P), diacyl glycerophosphoinositols (PI) and certain sphingolipids (SP). Other lipid representations include; glyerophosphoserines (PS); glycerophosphoethanolamines (PE); lyso PE (LPE); ceramides (Cer); galactosyl (Gal); sphinganines (sph); glycine lipids (GlyL); flavolipins (FL).

Mass spectrometry applications and analyses reveal that a diverse range of lipids are associated with gut resident representatives of *Bacteroides* (see Table S2 for a full list of the lipids identified in this study). We fully acknowledge that different lipids may be uncovered using other lipid extraction procedures. Multivariate analysis indicates that *B. vulgatus* and *B. fragilis* lipid signatures co-cluster, whilst *B. thetaiotaomicron* and *B. ovatus* lipid signatures occupy distinct positions (Figure 1A,B). This analysis suggests that *B. vulgatus* and *B. fragilis* may have similar lipidomes, which are distinct from both *B. thetaiotaomicron* and *B. ovatus*.

Targeted LC-MS–based lipidomics reveal the diversity of lipids present in *Bacteroides*. In total, we identified 170 individual lipids distributed across 4 lipid categories or 26 lipid ‘subgroups’ (according to convention defined in LIPID MAPS) (Figure 1C). The *Bacteroides* lipidome is dominated by glycerophospholipids (GP) and sphingolipids (SP), with smaller contributions from fatty acyls (FA) and glycerolipids (GL) (see Figure 1C). Targeted analysis also reveals that the observed differences in the lipid profile between *Bacteroides* species are largely driven by species-dependent signatures related to the presence of plasmalogens (P), diacyl glycerophosphoinositols (PI) and certain sphingolipids (SP), including dihydroceramidephosphoinositol (Cer PI) and α-galactosyl dihydroceramide (GalCer) (Figure 1D). In contrast, fatty acids, hydroxy fatty acids, *N*-acyl amines, diacylglycerol (DG), glycerophosphoethanolamine (both diacyl PE and lyso PE (LPE)), diacyl glycerophosphoserine (PS), dihydroceramide (dihydroCer) and dihydroCer phosphoethanolamines (Cer PE) represent relatively stable core lipids found in all species of *Bacteroides* examined in this study (Figure 1D).

### 3.2. N-Acyl Amines Comprise a Signifcant Proportion of the Fatty Acyl (FA) Component of Bacteroides Lipids

**Figure 2:**
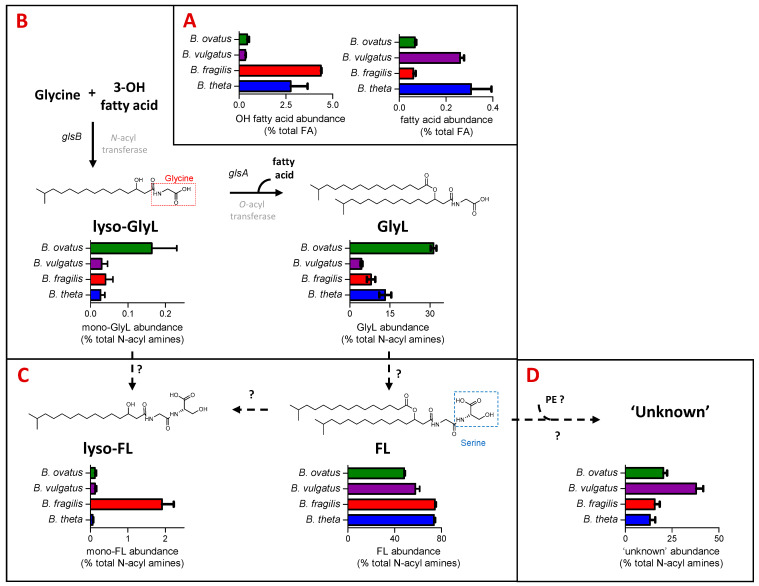
***N*-acyl amines comprise a significant proportion of the fatty acyl (FA) component of *Bacteroides* lipids, with some clear quantitative differences observed between species**. Here, we relate the actual lipid abundances to possible *Bacteroides* biochemical and genetic pathways towards their production while indicating the genetic gaps in our knowledge. (**A**) Relative abundance (expressed as % total FA) of hydroxy fatty acids and fatty acids among the four *Bacteroides* species tested. Hydroxy fatty acids and fatty acids are the building blocks to more complex *Bacteroides* lipids, including *N*-acyl amines, shown in (**B**–**D**). (**B**) Relative abundance (expressed as % total *N*-acyl amines) of mono- (lyso-GlyL) and diacylated glycine lipids (GlyL) generated from *N*- and *O*- acyl transferases encoded by characterised *glsB* and *glsA* genes, respectively [25], but shown here for four *Bacteroides* species. (**C**) Relative abundance (expressed as % total *N*-acyl amines) of mono- (lyso-FL) and diacylated flavolipins (FL) for four *Bacteroides* species. The exact biosynthesis of FL is unknown; hypothetically, they could be synthesised from the respective glycine lipid precursors by attaching serine to the terminal glycine moiety as proposed for *Porphyromonas gingivalis* [18,19]. (**D**) Relative abundance (expressed as % total *N*-acyl amines) of the ‘unknown’ lipids that we predict as generated from FL and glycerophosphoethanolamines (PE) for four *Bacteroides* species.

The FA category of LIPID MAPS includes fatty acids and *N*-acyl amines. *B. thetaiotaomicron* and *B. fragilis* have the highest representation in the FA category, which includes hydroxy fatty acids (C15 to C17) and *N*-acyl amines (Figure 1C). *N*-acyl amines comprising GlyL and FL with varying acyl chain lengths and their respective mono-acylated derivatives (mono-GlyL, mono-FL) were detected in all four *Bacteroides* species (see Figure 2).

GlyL represented between 4% of *B. fragils* and 32% of *B. ovatus* total *N*-acyl amines detected (Figure 2C). For the most part, the major GlyL was the previously reported GlyL at *m*/*z* 568 in the positive ion mode; however, *B. ovatus* contained similar amounts of a different glycine lipids at *m*/*z* 554 in the positive ion mode, presumably with a shorter carbon chain length (Figure S1B). For the most part, lyso-GlyL (also known as commendamide) was a minor component of the lipid signatures detected for all *Bacteroides* species examined (Figure 2C).

In general, FL accounted for most of the *N*-acyl amines detected (Figure 2D), and this lipid was abundantly represented amongst all *Bacteroides* species, with lyso-FL approximately 20 times more abundant in the *B. fragilis* lipid signature (Figure 2D). The best characterised FL, at *m*/*z* 655 in the positive ion mode, is also known as Lipid 654 (FL-654), owing to its molecular weight in the negative ion mode, and this was the most abundant *N*-acyl amine in extracts from *B. fragilis* and *B. vulgatus*, whilst the shorter carbon chain length FL, at *m*/*z* 641 in the positive ion mode, was the most abundant in *B. thetaiotaomicron* and *B. ovatus* (Figure S1A). These different chain lengths may indicate important strain-specific differential signalling potential [30].

In addition, a series of ‘unknown’ but predicted *N*-acyl amines with *m*/*z* values (in positive ion mode) of 1230.9207 (Unknown_1231), 1244.9363 (Unknown 1245), 1258.9520 (Unknown 1259) and 1272.9676 (Unknown_1273) were also detected (see Table S2). *B. vulgatus* contained a relatively higher proportion of these ‘unknown’ lipids (Figure 2E), with Unknown_1259 and Unknown_1273 as most abundant (Figure S1C). Therefore, the profile of *N*-acyl amines is qualitatively similar across all of the examined *Bacteroides*, although there are some quantitative differences that may be physiologically important, given the important signaling role of the glycine lipid family.

### 3.3. Dihydroceramide Phophoethanolamine (Cer PE) Is the Most Abundant Sphingolipid (SP) Detected in All Four Bacteroides

**Figure 3:**
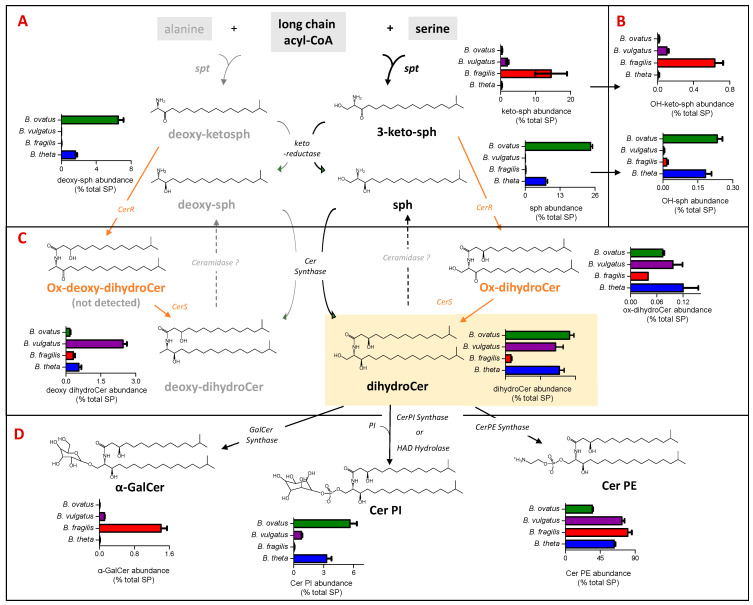
**Dihydroceramide phosphoethanolamine (Cer PE) is the most abundant sphingolipid (SP) detected in all four *Bacteroides* species**. The schematic indicates the relative representation of each lipid species and the proposed genetic and biochemical pathways contributing towards their production. (**A**) Relative abundance (expressed as % total PS) of sphingoid bases, which include keto-sphinganines (keto-sph) and sphinganines (sph) derived from either serine or alanine (deoxy derivatives) among the four *Bacteroides* species. (**B**) Relative abundance (expressed as % total SP) of hydroxy keto-sph and sph; exact position of hydroxylation is unknown. (**C**) Relative abundance (expressed as % total SP) of ceramide (Cer) lipids among the four *Bacteroides* species derived from either serine (dihydroCer) or alanine (deoxy-dihydroCer). (**D**) Relative abundance of complex *Bacteroides* SP (expressed as % total SP), including α-galactosyl dihydroCer (α-GalCer) and the phosphosphingolipids dihydroCer phosphosethanolamines (Cer PE) and dihydroCer phosphoinositols (Cer PI).

In this study, SP was found to represent between 19% *(B. ovatus*) and 29% (*B. vulgatus*) of the total lipids detected (Figure 2A). Figure 3 shows the levels of SP subgroups extracted from each examined *Bacteroides* species with respect to the steps in the SP biosynthesis pathway. Briefly, the biosynthesis of bacterial SP is initiated by serine palmitoyltransferase (SPT), which catalyses a reaction between a fatty acyl-CoA and serine, or alternatively alanine, to form keto-sphinganine (sph) or deoxy-keto-sph, respectively. The pathway to dihydroCer synthesis may proceed similarly to that observed in eukaryotes, harnessing keto reductase activity [31] to form sph. Alternatively bacterial Cer synthase (CerS) could directly add an acyl chain to 3-keto-sph to form an oxidised dihydroCer intermediate (ox-dihydroCer), which could then be reduced to dihydroCer by bacterial Cer reductase (CerR) [32]. DihydroCer represents the central hub of SP metabolism, and it can undergo modification with different head groups (Figure 3D). The biosynthesis of *B. fragilis* α-GalCer was recently reported via a ceramide UDP-GalCer synthase [33], whilst the biosynthesis of Cer PI was recently proposed through either Cer PI synthase or haloalkanoate dehalogenase (HAD) hydrolase activity [34].

In our study, dihydroCer was shown to account for approx. 20% of the SP fraction in *B. ovatus, B. vulgatus* and *B. thetaiotaomicron* (Figure 3C). However, in *B. fragilis*, dihydroCer accounted for only 2% of the SP fraction (Figure 3C). Instead, *B. fragilis* appears to accumulate higher levels of keto-sphinganines (keto-sph), which are upstream intermediates in the SP biosynthetic pathway (Figure 3A). Moreover, there were detectible levels of sphinganine (sph) and deoxy-sph in both *B. thetaiotamicron* and *B. ovatus* (Figure 3A). These intermediates may be generated by the reduction of keto-sph by a keto-reductase and/or the hydrolysis of dihydroCer by ceramidases (Figure 3A,C). Recent studies have suggested that there may be multiple pathways for SP biosynthesis in *Bacteroides*, and the varying levels of intermediates detected across the four species examined in this study support this observation [31,32].

Cer PE was identified as the most abundant SP in all four species, with levels between 35% for *B. ovatus* and 81% for *B. fragilis* of the total SP detected (Figure 3D). In the current study, Cer PI represented 3%, 6% and 1% of the total SP fraction in *B. thetaiotaomicron*, *B. ovatus* and *B. vulgatus*, respectively, and this SP was not detected in *B. fragilis* (Figure 3D). Moreover, α-GalCer was detected in only *B. fragilis* and *B. vulgatus* (Figure 3D). Therefore, there is a wide range (both qualitatively and quantitatively) of SP and their intermediates produced across the *Bacteroides* species examined in this study.

### 3.4. Plasmalogens and Phosphoinositol (PI) Lipids Are Not Found in All Bacteroides Species

**Figure 4:**
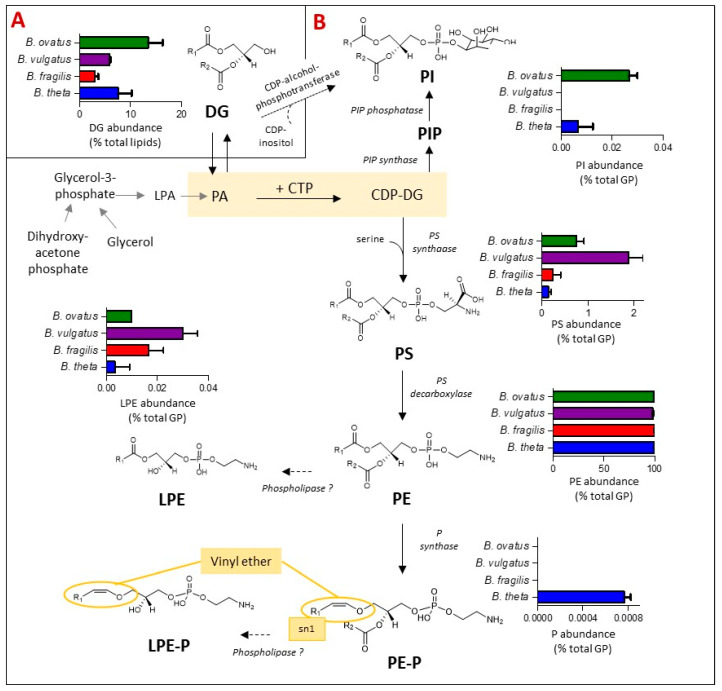
**The presence of plasmalogens and phosphoinositol (PI) lipids is dependent on the *Bacteroides* species**. The schematic indicates the relative representation of each lipid species and the proposed genetic and biochemical pathways contributing towards their production. (**A**) Relative abundance (expressed as % total lipid) of diacylglycerols (DG) for the four *Bacteroides* tested. DG act as metabolic intermediates in the biosynthesis of *Bacteroides* glycerophospholipids (GP). (**B**) Relative abundance (expressed as % total GP) of glycerophosphoinositol (PI), glycerophosphoserine (PS), glycerophosphoethanolamines (PE), mono-acylated or lyso PE (LPE) and the vinyl-ether containing plasmalogens (P), glycerophosphoethanolamines plasmalogen (PE-P) and lysophosphatidylethanolamine *plasmalogen* (LPE-P). Other abbreviations are: cytidine diphosphate-DG (CDP-DG); PI phosphate (PIP); glycerophosphoethanolamines plasmalogen (PE-P); PA: lycerophosphatic acid; lyso PA (LPA).

Glycerophospholipids (GP) are the primary buiding blocks of bacterial cell membranes. Their synthesis, biochemical diversity and relative levels amongst *Bacteroides* species were examined and are depicted in Figure 4. Amongst other bacteria representatives, the biosynthesis of most bacterial GP primarily begins from the central metabolite cytidine diphosphate-DG (CDP-DG), leading to the production of phosphoinositol (PI) in one direction or glycerophosphoserine (PS) in the other. PI is formed either via PI phosphate (PIP) intermediates [34,35] or directly via diacylglycerols (DG) and CDP-alcohol-phosphotransferase, the latter pathway is predicted *in silico* and it remains to be validated [34]. PS acts as a metabolic intermediate; it can undergo decarboxylation to form glycerophosphoethanolamines (PE) via PS decarboxylase [35]. In bacterial membranes, Lyso GP such as LPE are generated as metabolic intermediates in phospholipid synthesis or from membrane degradation via the action of phospholipases [36], whilst glycerophosphoethanolamines plasmalogen (PE-P) can be formed from PE via plasmalogen synthase (P synthase) in anaerobes, specifically *Clostridium perfringins* [37].

Bringing these systems and representations together for *Bacteroides* species (Figure 4), DG, the simplest glycerol based membrane lipid and a metabolic intermediate to GP, accounts for between 3% (*B. fragilis*) and 13% (*B. ovatus*) of the total lipid content of *Bacteroides* (Figure 4A) under anaerobic conditions. Thereafter, the GP lipid fraction was dominated by diacyl PE (97% of total GP) in all four *Bacteroides* species tested (Figure 4B), with minor amounts of lyso-PE (LPE) and diacyl PS also detected (Figure 4B). On the other hand, diacyl PI was detected in lipid extracts of *B. thetaiotaomicron* and *B. ovatus* but not in *B. fragilis* and *B. vulgatus* (Figure 4B).

Interestingly, we detected a number of plasmalogens (both diacylated (PE-P) and lyso-derivatives (LPE-P)) in *B. thetaiotaomicron* only (Figure 4B) at *m*/*z* [M-H]- of 576.4035, 590.4191, 604.4348, 618.4504, 632.4661, 646.4817, 660.4974 (PE-P) and 394.2364, 408.2521, 422.2677, 436.2834 (LPE-P) (Table S2). Fragments at *m*/*z* 196.0380 and 140.0118, corresponding to the loss of the plasmenyl group and the ethanolamine phosphate ion, respectively, were detected in negative mode MS2 spectra, confirming that these plasmalogens are derived from PE. The presence of plasmalogens in *B. thetaiotaomicron* was confirmed by specific acid hydrolysis assay (see Figure S2).

### 3.5. A Mutation in Sphingolipid (SP) Biosynthesis Results in Global Changes in the Lipid Signature, including Reductions in the Levels of GlyL

**Figure 5:**
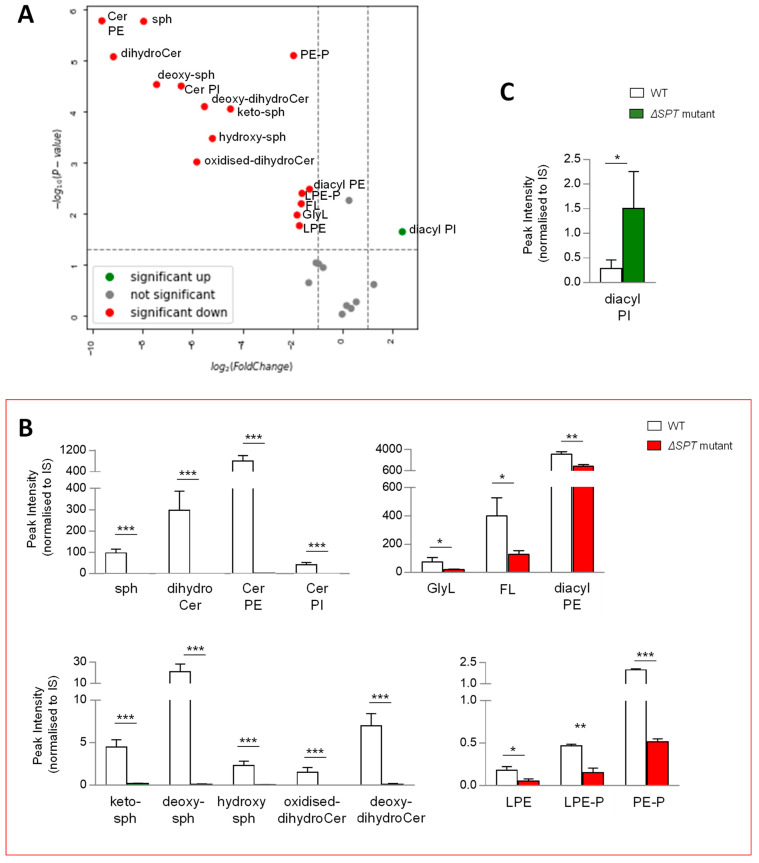
**A mutation in sphingolipid (SP) biosynthesis (Δ*SPT* mutant) results in significant changes in the lipid signature of *B. thetaiotaomicron.*** (**A**) Univariate analyses (volcano plot) reveal significant changes (>2 fold, t-test *p*-value < 0.05) to the lipid profile of *B. thetaiotaomicron;* 15 of the 26 lipid ‘subgroups’ were significantly reduced (depicted in red) in the Δ*SPT* mutant relative to the WT parent, 1 ‘subgroup’ was significantly increased (depicted in green), whilst 10 ‘subgroups’ were either unchanged or not detected (depicted in grey). (**B**) The SP ‘subgroups’ detected in WT *B. thetaiotaomicron* were completely depleted in Δ*SPT* mutant extracts. Significantly decreased lipids were glycine lipids (GlyL) and flavolipins (FL) as well as both lyso- (L) and di- (diacyl) acylated glycerophosphoethanolamines (PE) and their respective plasmalogens (P). (**C**) Diacyl glycerophosphoinoistols (PI) were the only significantly decreased lipid ‘subgroup’ related to Δ*SPT* mutation in *B. thetaiotaomicron.* All data shown are represented as mean values from n = 3 independent experiments. Standard deviation (SD) from the mean is indicated. Student T test was applied: *** *p* < 0.001, ** *p* < 0.005, * *p* < 0.05 to log transformed data prior to univariate analyses. The data shown represent area under the curve, untransformed peak intensity measures.

Given that the synthesis of all lipids requires similar building blocks, we reasoned that disrupting the production of any class of lipids could result in major compensatory lipid signature alterations. To qualify and measure these changes, we compared the lipid signatures of *B. thetaiotaomicron* ∆*SPT*, mutated for the gene encoding serine palmitoyltransferase, the enzyme required for the first step in SP biosynthesis, with the wild-type parent strain, post anaerobic growth and lipid extraction at 1OD_600_ biomass. Following extraction, mass spectrometry and analysis, as described above, multivariate analyses and comparison of lipid signatures showed that deletion of *SPT* results in dramatic changes to the *B. thetaiotaomicron* lipid profile (Figure 5A and Figure S3C). Moreover, these changes were not limited to SP lipids, indeed, 15 of the 26 lipid ‘subgroups’ (Figure 5A) or 86 of 170 individual lipids (Figure S3C) were significantly decreased in the *∆SPT* mutant compared to the wild-type parent strain (>2 fold, *p* value (<0.05)). Furthermore, in the *∆SPT* mutant strain, all SPs were depleted, whilst GlyL and FL were also significantly decreased relative to the WT parent (Figure 5B). Moreover, all PE (mono- and diacyl) and PE plasmalogens (mono- and diacyl) were significantly reduced in the ∆*SPT* mutant (Figure 5B). Indeed, the total identifiable lipids detected in the ∆*SPT* mutant decreased by 60% overall relative to the WT (Figure S3B). Diacyl PI was the only lipid subgoup that increased in the ∆*SPT* mutant relative to WT (Figure 5C). Therefore, as predicted, a mutation in SP production results in global changes in the lipid profile of the membranes of *B. thetaiotaomicron*.

### 3.6. A Mutation in Glycine Lipid Biosynthesis Results in Changes in the Sphingolipid Pool

**Figure 6:**
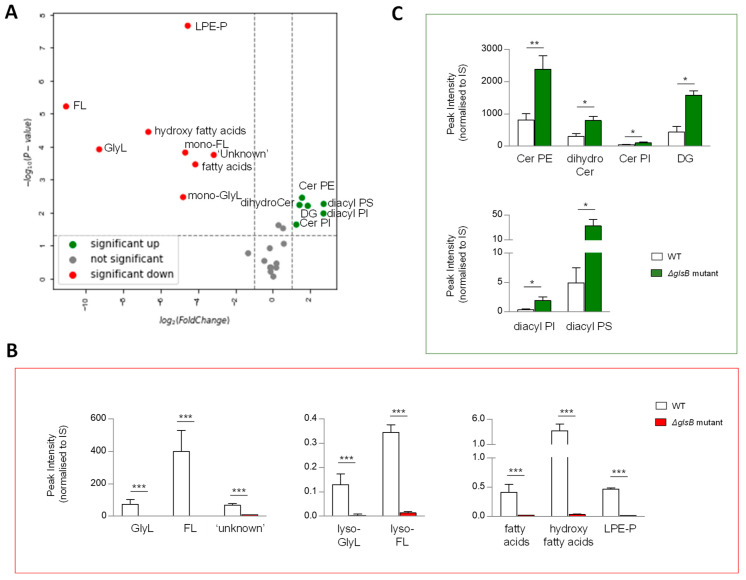
**A mutation in glycine lipid biosynthesis (Δ*glsB*) results in changes in the sphingolipid (SP) pool relative to wild type (WT) *B. thetaiotaomicron.*** (**A**) Univariate analyses (volcano plot) reveal significant (fold change >2 and t-test *p*-value < 0.05) changes to the lipid profile of *B. thetaiotaomicron* with 8 of the 26 lipid ‘subgroups’ significantly decreased (indicated in red) in the *B. thetaiotaomicron* Δ*glsB* mutant relative to the parent WT strain, while 6 were significantly increased (depicted in green) and 12 were either unchanged or not detected (ND) (depicted in grey). (**B**) Δ*glsB* resulted in complete depletion of Glycine lipids (GlyL) and flavolipins (FL). Other significantly decreased lipids were a series of ‘unknown’ lipids, which we predict as *N*-acyl amine lipids, fatty acids, hydroxy fatty acids and glycerophosphoethanolamines (PE) plasmalogens (P), (mono-acylated, LPE-P). (**C**) Many SP were significantly increased as a result of *glsB* mutation; they included dihydroceramides (dihydroCer), dihydroCer ethanolamines (Cer PE) and dihydroCer inositols (Cer PI). Significant increases were also observed for diacylglycerols (DG), diacyl glycerophosphoinoistols (PI) and diacyl glycerophosphoserines (PS). All data shown are mean values of n = 3 ± standard deviation from the mean (SD). Student T test was applied: *** *p* < 0.001, ** *p* < 0.005, * *p* < 0.05 to log-transformed data prior to univariate analyses. The data shown represent area under the curve, un-transformed peak intensity measures.

In *B. thetaiotaomicron*, the *glsB* gene encodes the enzyme responsible for the first step in glycine lipid biosythesis (Figure 2B) [25]. We reasoned that disrupting the enzyme activity may ilicit global changes in its lipid signature. To qualify and measure these changes, we compared the lipid signatures of *B. thetaiotaomicron* ∆*glsB* with the wild-type parent strain, post anaerobic growth and post lipid extraction of 1OD_600_ biomass. Following extraction, mass spectrometry and analysis, as described above, multivariate analyses and comparison of lipid signatures showed that 8 of the 26 lipid ‘subgroups’ or 36 of the 170 individual lipids identified in this study were decreased in the ∆*glsB* mutant compared to the WT strain (Figure 6A and Figure S4A–C). As expected, GlyL and the related FL and complex ‘unknown’ lipids were depleted in the ∆*glsB* mutant extracts compared to the WT strain (Figure 6B). On the other hand, 6 of the 26 lipid ‘subgroups’ (or 37 of the 170 individual lipids) proved significantly increased in the ∆*glsB* mutant compared to the WT parent strain (Figure 6C). These lipids include SP subgroups Cer PE, dihydroCer and Cer PI (Figure 6C) and GP subgroups DG, diacyl PI and diacyl PS (Figure 6C). Therefore, it appears that depletion of the glycine lipids is compensated for by increasing other lipid groups, particularily SP and GP.

## 4. Discussion

Microbial lipids are becoming recognised as interkingdom signalling molecules, and as such, they represent an interesting avenue for the potential development of novel biomarkers and/or therapeutics. In this study, we set out to construct a lipid map for a range of mammalian gut resident *Bacteroides* species, namely *B. thetaiotaomicron*, *B. fragillis*, *B.ovatus* and *B. vulgatus*. Individually, these selected *Bacteroides* may account for up to 6% of intestinal bacteria in healthy humans, with *B. vulgatus* reported as enriched to represent up to 40% in patients with Crohn’s Disease [38]. This work points to the presence of core lipid species, common to all *Bacteroides* species examined, but also to species-specific lipid signatures, which could be disrupted and redistributed through targeted pathway mutation. We acknowledge that results may have differed if other lipid extraction procedures would have been used.

Those identified represented four LIPID MAPS categories and 26 lipid ‘subgroups’ (Table S2) across the four selected species. PE (diacylated) of various fatty acyl chain lengths (Table S2) was noted as the major core lipid subgroup in all four *Bacteroides* species examined (Figure 4). Very recently, Bae et al. [30] identified a diacyl PE, with two branched fatty acyl chains (PE 15:0a/15:0i), from the cell membrane of *Akkermansia muciniphila*, as an agonist of TLR2-TLR1, which leads to the release of certain cytokines. This immunomodulatory activity is dependent on the presence of methyl branches on both fatty acyl chains; also, there is a requirement for one fatty acyl chain as antesio-branched while the other is iso-branched. It is possible that one of the three PE (30:0) isomers, detected in the current study (Table S2), may contain a similar molecular structure.

Our data suggest that PE is likely formed via the decarboxylation of PS, given that PS (diacylated) was also detected, albeit in relatively minor amounts, in all four species. Different lyso-PE (LPE) species were also noted as minor core lipids in all four *Bacteroides.* These lipids are likely generated as metabolic intermediates in PE synthesis or from the degradation of PE [36]. The role of LPE in bacteria remains poorly characterised, although it is believed that LPE may mitigate membrane stress induced by non-bilayer lipids such as cardiolipin. Whilst CL is an important GP in some Gram-negative bacteria, such as *E. coli* [35], and CL has been reported in some *Bacteroides* [39,40], we did not detect CL in any of the *Bacteroides* species examined in this study.

Plasmalogens, or vinyl ether lipids, are produced by the modification of the fatty acid at the sn-1 position of GP such that it is linked via an alkenyl or plasmenyl bond rather than an ester bond (Figure 3 shows the structure of PE Plasmalogen). Plasmalogens have a broad phylogenetic distribution; they are present in biological membranes of bacteria, protozoa, invertebrates and mammals [41]. Amongst bacteria, plasmalogens are rarely detected in aerobes [42]; they are sparse in facultative anaerobes [43] but appear to be common in anaerobes including certain gut-associated *Bifidobacteria* and *Clostridia* species [43,44,45,46,47,48]. Interestingly, in our study, plasmalogens were detected in one species of *Bacteroides* only, *B. thetaiotaomicron* (Figure 4), suggesting that these lipids are not ubiquitous in this genus. In 1969, Kamio et al. [48] reported the occurence of plasmalogens in strict anaerobe *Bacteroides ruminicola*, isolated from the rumen of a sheep; the strain has since been reclassified as *Prevotella ruminicola* species, genus *Fibrobacter.* Hence, our study is the first to confirm the presence of PE plasmalogens, both mono and diacyl, in a species of *Bacteroides* that is a normal component of the human gut microbiota. In mammals, plasmalogens play unique roles in membrane structure; in membrane trafficking and in cell signalling, reduced levels of circulating plasmalogens are linked to metabolic and neurological diseases including diabetes and Alzheimer’s disease [49,50,51]. The exact role(s) of plasmalogens in and from bacteria are not yet known; however, by virtue of their vinyl ether bond, they are likely to be involved in modulating membrane morphology, with the potential to provide protection from oxidative stress [52].

We previously described a family of *N*-acyl amines in *B. thetaiotaomicron*, called glycine lipids [25]. In this study we confirmed the presence of glycine lipids in all species of *Bacteroides* tested, suggesting that these lipids are widespread in this genus (Figure 2). Indeed, bioinformatic analysis indicates that the genetic potential for the production of glycine lipids is restricted to genera in the phylum Bacteroidota (our unpublished data). Nonetheless, there were species differences in the relative quantities of specific glycine lipids, despite identical and controlled growth and lipid isolation conditions. The physiological relevance of these differences is not clear. FL-654 was the most abundant glycine lipid detected in all *Bacteroides* tested. FL-654 has been reported to act as a TLR2 agonist in several studies, suggesting a possible role in inflammation in the host [14,17,18]. More recently, low-density lipoprotein receptor deficient (Ldlr−/−) mice fed a high fat diet (HFD), but who received chronic, 7-week, intraperitoneal administration of FL-654, were attenuated for atherosclerosis progression, and they displayed decreased markers of liver injury compared with vehicle control-injected mice [53], suggesting that these lipids may be beneficial. The glycine lipid family also includes high-molecular lipid molecules such as Lipid 1256, characterized from *Porphyromonas*, a relative of *Bacteroides*, consisting of a diacyl glycerophosphoglycerol (PG) linked to the FL [19]. Lipid 1256 was reported to be even more potent as a TLR2 ligand than the related GlyL and FL [19]. In the current study, we initially assumed that Unknown_1259 (Table S2) was the same as Lipid 1256; however, given the absence of PG in *Bacteroides* lipid extracts and the difference of a proton in the observed *m*/*z*, Unknown_1259 is more likely to be FL linked to PE. Work is ongoing to confirm the structure of this potentially novel lipid.

Compared to well-studied bacteria such as *E. coli*, *Bacteroides* do have an unusual membrane lipid composition, in that approximately 50% of the lipids, extractable with chloroform-methanol, are SP or free ceramides [54]. In the present study, using isopropanol extraction, SP represented between 19% and 29% of the total lipids detected in *B. ovatus and B. vulgatus*, respectively. For the most part, dihydroCer and Cer PE (dihydroCerPE in bacteria) represent the core SP detected (Figure 3), and this is typical of bacteria in the phylum *Bacteroidota* [55]. Interestingly, both dihydroCer and Cer PE are shown to be negatively correlated with inflammation and with Inflammatory Bowel Disese (IBD) in humans [13]. The same authors also reported that deoxy-dihydroCer in *B. thetaiotaomicron* is formed via the utilisation of alanine, rather than serine, by SPT. In the present study, putative deoxy-dihydroCer was detected in all four *Bacteorides* species tested, and it was notably higher in representation in *B. vulgatus* (Figure 3). In addition, a putative oxidised dihydroCer (ox-dihydroCer) lipid was detected as a minor core lipid, supporting the notion that dihydroCer synthesis may proceed via bacterial ceramide synthase (CerS), which could directly add an acyl chain to keto-sph producing ox-dihydroCer, which may then be reduced to dihydroCer by bacterial ceramide reductase (CerR) (Figure 3). The upstream SP, keto-sph, was particularily abundant in *B. fragilis*, suggesting a potentially slower conversion to dihydroCer and/or faster rate of sythesis via SPT (Figure 3A). This highlights potentially important species-specific differences in enzyme kinetics and flux, through the SP biosynthetic pathway. The presence of sph and deoxy-sph in *B. thetaiotaomicron* and *B. ovatus* may be indicative of increased keto-reductase activity [30] and/or a slower rate of conversion to dihydroCer in these *Bacteroides* species. It may also be interpreted to indicate the presence of a ceramidase, which hydrolyses dihydroCer to sph [32], an activity that appears absent in both the *B. fragilis* and *B. vulgatus* species examined.

Depending on the species, two other complex SP were detected in the present study. Cer PI, which consist of a inositol phosphate on a sphingoid backbone, were detected in *B. thetaiotaomicron* and *B.ovatus* as reported for *B. thetaiotaomicron* previously by Brown et al. [13]. Here, we also show that Cer PI are present in *B. vulgatus* (Figure 3). The gene clusters reponsible for inositol lipid synthesis in some *Bacteroides* species have recently been described [13,34,56] and involve either PI Cer synthase, typical of *B. thetaiotaomicron* and *B.ovatus*, or HAD hydrolase activity, typical of *B. vulgatus* (Figure 3). Heaver et al. [34] report that inositol and inositol lipids, both membrane and capsule lipids, are likely implicated in resistance to host immune defences and therefore may influence their fitness and maintenance in the mammalian gut. The ‘non-phosphate’ containing complex glycosphingolipids, α-GalCer, were detected in *B. fragilis* and, to a lesser extent, in *B. vulgatus*. This is consistent with previous reports on the generation of α-GalCer by *B. fragilis* [10,56] and lower levels produced by *B. vulgatus* [57]. *B. fragilis* α-GalCer was recently reported as dependant on ceramide UDP-galactosylceramide synthase activity [33]. α-GalCer has been shown to be a potent stimulator for invariant NKT cells [10], whereby the sphinganine chain branching is a critical determinant of NKT activation [58].

SP and glycine lipids are found in all *Bacteroides* tested and are likely to have a structural role in the membrane of these bacteria. Therefore, we examined the changes in lipid signatures following mutations to these two major bioactive lipiid pathways in *B. thetaiotaomicron*. Using a mutation in the *glsB* gene, we showed that *B. thetaiotaomicron* compensate for the absence of glycine lipids by increasing some SP, DG, diacyl PI and diacyl PS. In contrast, we show an overall decrease in lipid diversity associated to the *B. thetaiotaomicron* ∆SPT mutant, including a significant decrease in many glycine lipids, PE (both diacyl and lyso) and PE plasmalogens (both diacyl and lyso). The only attempt at compensation appears to be through a signifcant increase in PI (Figure 5C). Given that Cer PI are not formed in the ∆SPT mutant, the increase in PI may simply be due to their reduced coupling to ceramide (Figure 3D). Thus, perturbations in glycine lipid or SP biosynthesis results in significant and distinct changes in the levels of other lipids, suggesting the existence of compensatory mechanisms required to maintain the functionality of the bacterial membrane. The relatively dramatic global lipid decreases in the ∆SPT mutant may suggest that SP have a key structural role in the membranes of *Bacteroides*; without SP, other key membrane lipids may be impeded from organising or inserting into the membrane such that they are therefore depleted from the lipidome.

In summary, we show that *Bacteroides* produce diverse lipids, some of which are core lipids while others are species specific. We point to the biochemical processes and to the gaps in our knowledge in understanding their production under anaerobic conditions. We further demonstrate that plasmalogen production is unique to *B. thetaiotaomicron* among the species examined. The exact role of these plasmalogens, in or between bacteria, in reacting to oxygen or as molecular signalling molecules to the host, remains to be elucidated. Given plasmalogen representation in key gut resident bacteria, their modulation may represent untapped therapeutic targets for different disease states [59]. For *B. fragilis*, the most notable difference in the lipid profile was their relatively higher abundance of lyso-FL keto-sph and α-GalCer. There is a sparsity of knowledge on the bio-activity of bacterially derived keto-sph. The bioactivity of α-GalCer, however, has received considerable attention, since certain lyso-FL species can act as agonists of TLR2 [10,56,58], a bacterial recognition Toll-like receptor that mediates macrophage release. *B. ovatus* was notable—relative to the other species—in its accumulation of DG, PI and Cer PI. Indeed, *B. ovatus* ATCC8483 has been shown to reduce mucosal inflammation by up-regulating IL-22 secretion [60,61]. Interestingly, *B. vulgatus* presented relatively more ‘unknown’ lipids, specifically LPE and PS and deoxy-dihydroCer. Given the association of *B.vulgatus* with IBD [15], these observations may prove important. The ‘unknown’ lipids are structurally related to Lipid 1256, a potent TLR2 ligand that also promotes the production of pro-inflammatory cytokines. In addition, deoxy-dihydroCer are considered ‘dead-end’ toxic lipids [62], and they are implicated in the progression of Type 2 diabetes [63,64].

In short, this study aimed to elucidate, understand, characterize and examine the relative synthesis and lipid signatures associated with important gut resident bacteria, *Bacteroides* species. It unearthed key and unique lipid species representation. It will provide a useful platform for further studies to elucidate lipid-based host–microbe and microbe–microbe dialogues and may prove important in the context of addressing host health and disease states.

## Data Availability

The data presented in this study are openly available in [EMBL-EBI MetaboLights database] at [10.1093/nar/gkz1019], reference number [MTBLS7318].

## Supplementary Materials

The following supporting information can be downloaded at: https://www.mdpi.com/article/10.3390/metabo13030360/s1, Figure S1: The relative abundance of each Flavolipin (FL, A), glycine lipid (GLyL, B) and ‘unknown’ lipid (C) for each *Bacteroides* species examined.; Figure S2: The presence of plasmalogens is confirmed in *B. thetaiotaomicron* extracts; Figure S3: Effect of *SPT* mutation on the lipid profile of *B. thetaiotaomicron*; Figure S4: Effect of *glsB* mutation on the lipid profile of *B. thetaiotaomicron*; Table S1: Chromatographic gradient applied for lipid profiling; Table S2: Lipids detected by LC-MS in *Bacteroides* isopropanol extracts.
