# Supplementary material for: Lipidomic Analysis Reveals Differences in Bacteroides Species Driven Largely by Plasmalogens, Glycerophosphoinositols and Certain Sphingolipids
Source: Metabolites. 2023 Feb 28;13(3):360. doi: 10.3390/metabo13030360 (PMC10056535; doi:10.3390/metabo13030360)
Supplement: Supplementary file 1 [file metabolites-13-00360-s001.zip › metabolites-2191894-supplementary.pdf]

Supplementary Information:

Lipidomic analysis reveals differences in *Bacteroides* species driven largely by plasmalogens, glycerophosphoinositols and certain sphingolipids

Eileen Ryan,[a,b] Belén Gonzalez Pastor,[a,c] Lee A. Gethings,[d,e,f] David J. Clarke,[a,c]* Susan A. Joyce[a,b]*

[a]APC Microbiome Ireland, University College Cork, Ireland

[b]School of Biochemistry & Cell Biology, University College Cork, Ireland

[c]School of Microbiology, University College Cork, Ireland

[d]Waters, Stamford Avenue, Altrincham Road, Wilmslow, SK9 4AX, UK

[e]Division of Infection, Immunity and Respiratory Medicine, Faculty of Biology, Medicine and Health, Manchester Institute of Biotechnology, University of Manchester, Manchester, M1 7DN, UK

[f]Faculty of Health and Medical Sciences, University of Surrey, Guildford, Surrey, GU2 7XH, UK

*Corresponding authors, contributed equally to this manuscript

Supporting Information

Table SI. Chromatographic gradient applied for lipid profiling

Time (min)	% A	% B	Curve
Initial	60	40	Initial
2.0	57	43	6
2.1	50	50	1
12.0	46	54	6
12.1	30	70	1
18.0	1	99	6
18.1	60	40	6
20	60	40	1

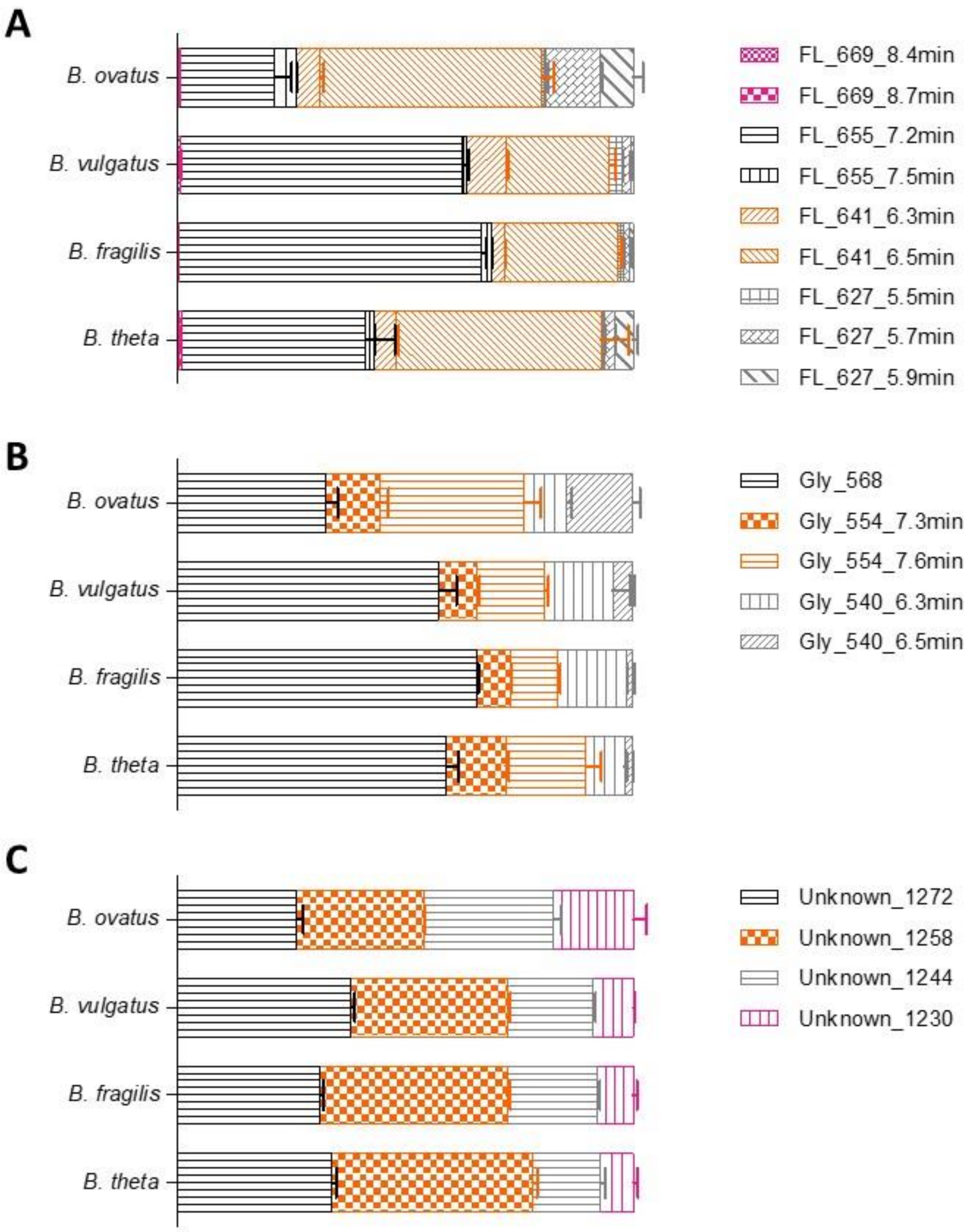

Figure S1: The relative abundance of each Flavolipin (FL, A), glycine lipid (GLyL, B) and 'unknown' lipid (C) for each *Bacteroides* species examined.

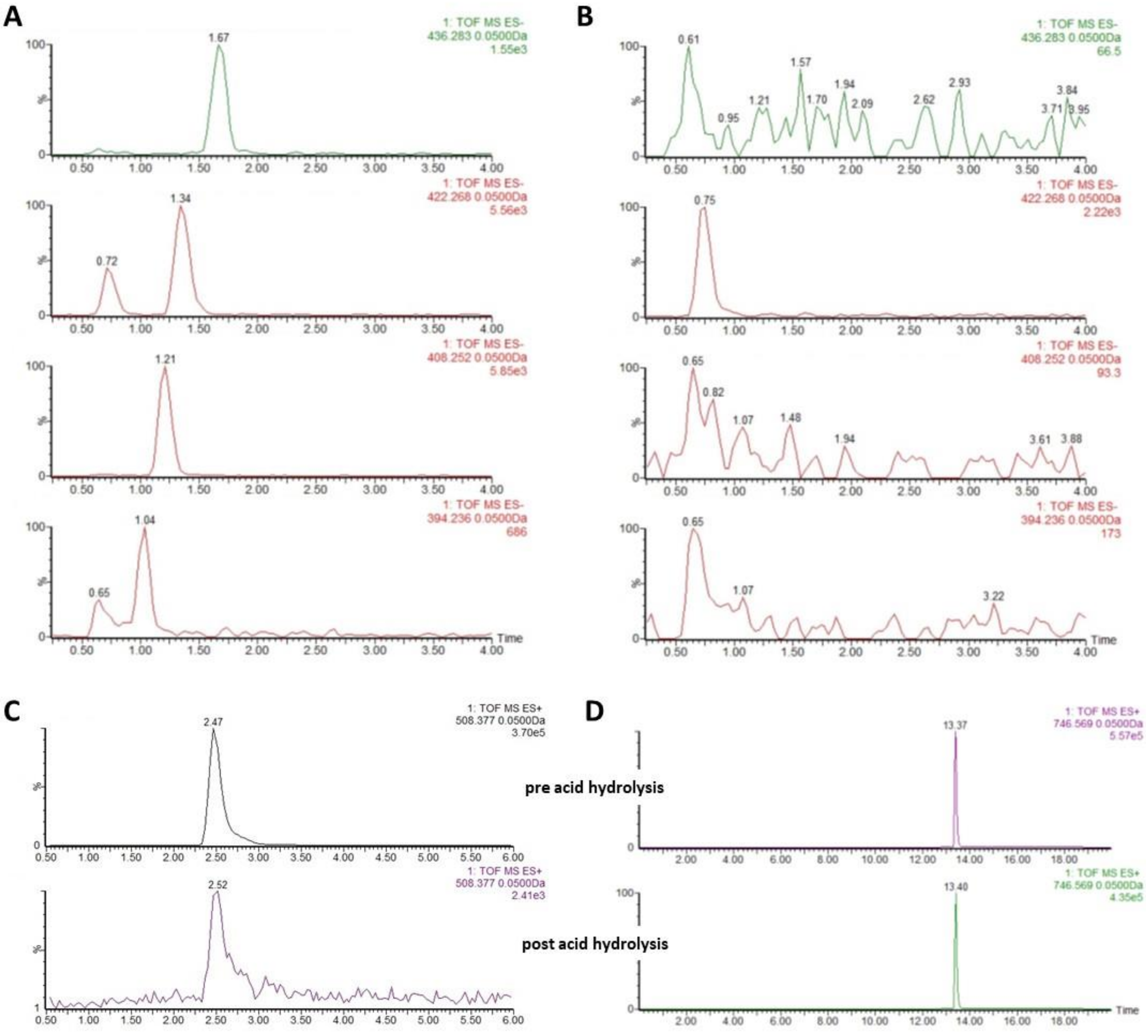

Figure S2. The presence of plasmalogens is confirmed in *B. thetaiotaomicron* extracts Chromatograms show (A) Presence of lyso-PE (LPE) plasmalogens (P) in *B. thetaiotaomicron* wild-type extracts at t_R 1.04 (LPE (P-13:0)), 1.21 (LPE (P-14:0)), 1.34 (LPE (P-15:0)) and 1.67 (LPE (P-16:0)) mins in the absence of acid hydrolysis (B) Absence of LPE-P in *B. thetaiotaomicron* wild-type extracts subjected to acid hydrolysis. (C) A positive control, lyso-glycerophosphocholine (LPC) P-18:0 was employed in acid hydrolysis experiments, in the absence of acid it was detected at 2.47 min and when subjected to acid hydrolysis LPE (P-18:0) was greatly reduced. A negative control, diacyl PE (PE 18:0/18:1) was employed in acid hydrolysis experiments, in the absence of acid it was detected at 2.4 min and when subjected to acid hydrolysis PE (18:0/18:1) was largely unaffected.

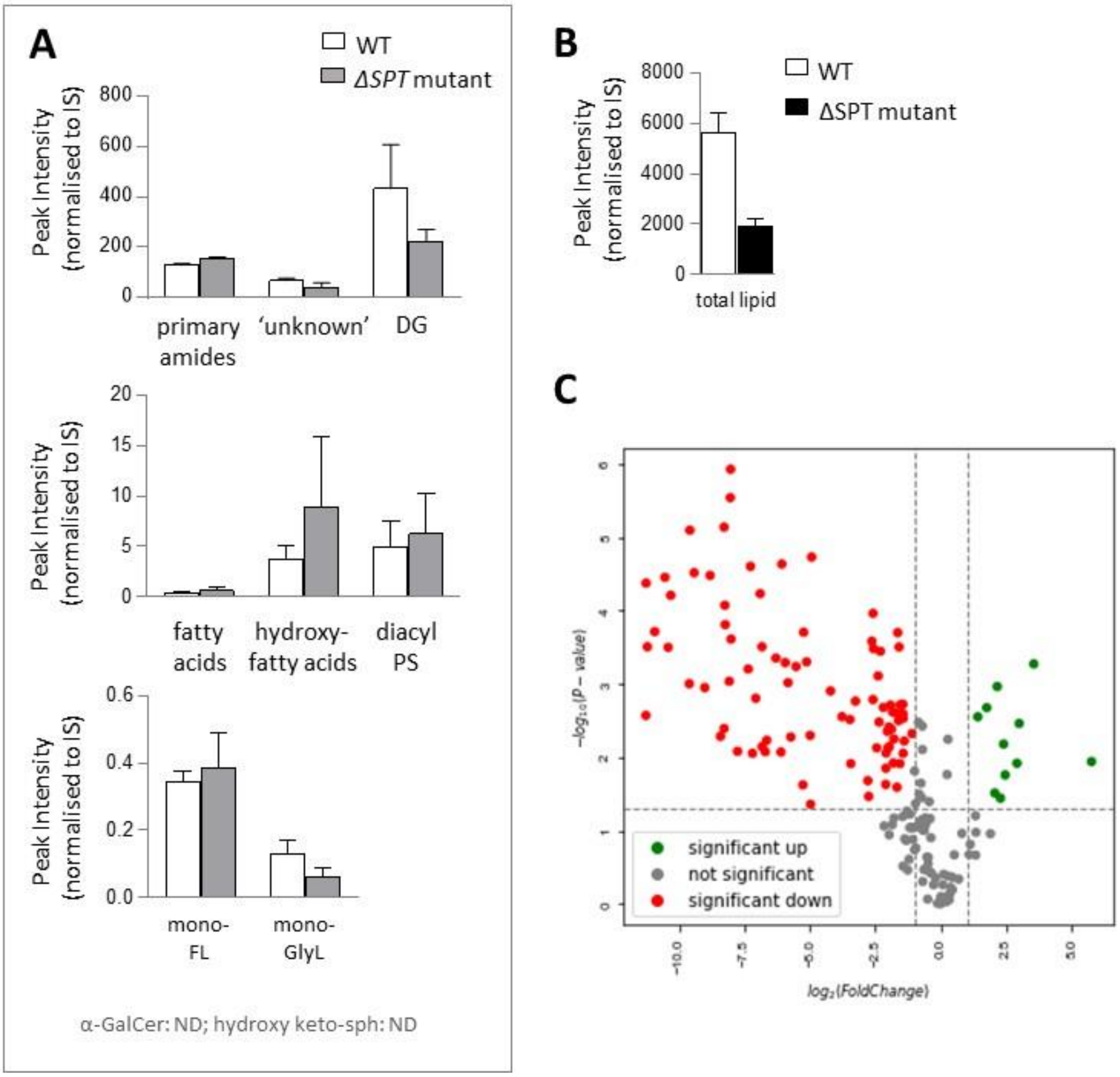

Figure S3. Effect of *SPT* mutation on the lipid profile of *B. thetaiotaomicron* (A) 10 of the 26 lipid subgroups were not significantly altered (as determined by both fold change > 2 and p-value <0.05) or not detected in *B. thetaiotaomicron ΔSPT* mutant versus wild-type (WT). (B) Mean total lipid profile (normalised to internal standard) of identified lipids in *ΔSPT* mutant versus WT extracts (C) Univariate analyses (volcano plot) reveal significant (fold change >2 and t-test p-value < 0.05) changes to the lipid profile of *B. thetaiotaomicron* whereby 86 of the 170 lipids were significantly reduced (red) in *B. thetaiotaomicron ΔSPT* mutant relative to the parent WT strain, 11 lipids were significantly increased (green) and 73 lipids were either unchanged or not detected (grey). All data shown are mean values of n=3 independent experiments ± standard deviation from the mean (SD). *** P < 0.001, ** P<0.005, P<0.05 and was log transformed prior to univariate analyses (data shown is un-transformed area under the curve peak intensity).

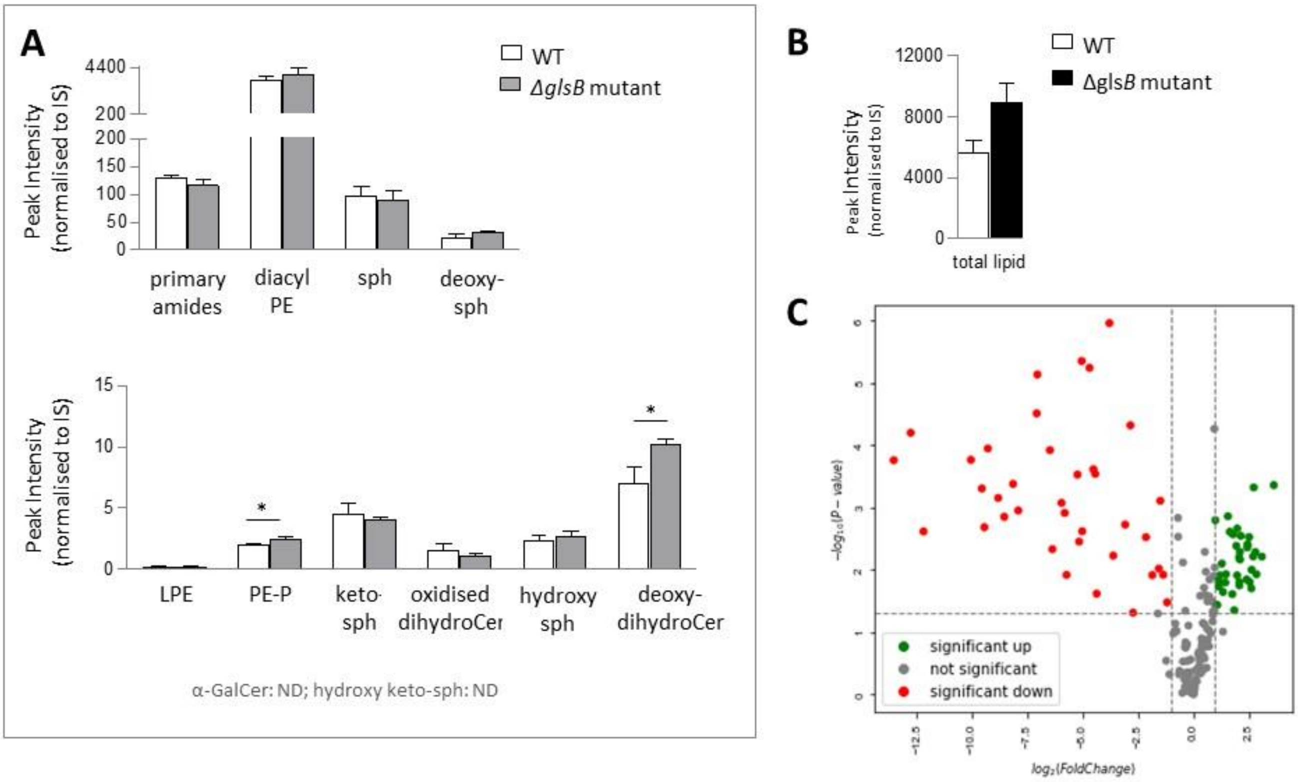

Figure S4. Effect of *glsB* mutation on the lipid profile of *B. thetaiotaomicron* (A) 12 of the 26 lipid 'subgroups' were either not significantly altered or not detected (ND) in *B. thetaiotaomicron ΔglsB* mutant versus wild-type (WT) (B) Mean total lipid profile (normalised to internal standard) of identified lipids in Δ*glsB* mutant versus WT extracts (C) Univariate analyses (volcano plot) reveal significant (fold change >2 and t-test p-value < 0.05) changes whereby 36 lipids were significantly reduced (red) in *B. thetaiotaomicron ΔglsB* mutant relative to the WT parent strain, 37 lipids were significantly increased (green) and 97 lipids were either unchanged or not detected (grey). All data shown are mean values of n=3 independent experiments ± standard deviation from the mean (SD). *** P < 0.001, ** P<0.005, P<0.05 and was log transformed prior to univariate analyses (data shown is un-transformed area under the curve peak intensity).

Table S2. Lipids detected by LC-MS in Bacteroide isopropanol extracts

Lipid	Formula	Ion	m/z	t_R	Lipid Category	Lipid Class	Lipid Subclass
DG (25:0)	C28H54O5	M+Na	493.3869	5.00	Glycerolipids (GL)	Diradylglycerols	Diacylglycerols (DG)
DG (26:0)	C29H56O5	M+Na	507.4025	5.50	Glycerolipids (GL)	Diradylglycerols	Diacylglycerols (DG)
DG (27:0)	C30H58O5	M+Na	521.4182	6.60	Glycerolipids (GL)	Diradylglycerols	Diacylglycerols (DG)
DG (28:0)_7.3min	C31H60O5	M+Na	535.4338	7.26	Glycerolipids (GL)	Diradylglycerols	Diacylglycerols (DG)
DG (28:0)_7.6min	C31H60O5	M+Na	535.4338	7.62	Glycerolipids (GL)	Diradylglycerols	Diacylglycerols (DG)
DG (28:0)_8.0min	C31H60O5	M+Na	535.4338	7.94	Glycerolipids (GL)	Diradylglycerols	Diacylglycerols (DG)
DG (29:0)_8.4min	C32H62O5	M+Na	549.4495	8.39	Glycerolipids (GL)	Diradylglycerols	Diacylglycerols (DG)
DG (29:0)_8.7min	C32H62O5	M+Na	549.4495	8.74	Glycerolipids (GL)	Diradylglycerols	Diacylglycerols (DG)
DG (30:0)_9.6min	C33H64O5	M+Na	563.4651	9.63	Glycerolipids (GL)	Diradylglycerols	Diacylglycerols (DG)
DG (30:0)_10.1min	C33H64O5	M+Na	563.4651	10.12	Glycerolipids (GL)	Diradylglycerols	Diacylglycerols (DG)
DG (30:0)_10.7min	C33H64O5	M+Na	563.4651	10.64	Glycerolipids (GL)	Diradylglycerols	Diacylglycerols (DG)
DG (31:0)_11.2min	C34H66O5	M+Na	577.4808	11.24	Glycerolipids (GL)	Diradylglycerols	Diacylglycerols (DG)
DG (31:0)_11.6min	C34H66O5	M+Na	577.4808	11.63	Glycerolipids (GL)	Diradylglycerols	Diacylglycerols (DG)
DG (32:0)_12.8min	C35H68O5	M+Na	591.4968	12.80	Glycerolipids (GL)	Diradylglycerols	Diacylglycerols (DG)
DG (32:0)_13.0min	C35H68O5	M+Na	591.4968	13.04	Glycerolipids (GL)	Diradylglycerols	Diacylglycerols (DG)
DG (32:1)	C35H66O5	M+Na	589.4811	10.94	Glycerolipids (GL)	Diradylglycerols	Diacylglycerols (DG)
DG (33:0)	C36H70O5	M+Na	605.5121	13.28	Glycerolipids (GL)	Diradylglycerols	Diacylglycerols (DG)
DG (34:0)	C37H72O5	M+Na	619.5277	13.67	Glycerolipids (GL)	Diradylglycerols	Diacylglycerols (DG)
DG (34:1)	C37H70O5	M+Na	617.5124	13.10	Glycerolipids (GL)	Diradylglycerols	Diacylglycerols (DG)
C13:0	C13H26O2	M-H-	213.1860	1.57	Fatty Acyls (FA)	Fatty Acids & Conjugates	Straight/Branched
C14:0	C14H28O2	M-H-	227.2018	1.94	Fatty Acyls (FA)	Fatty Acids & Conjugates	Straight/Branched
C15:0	C15H30O2	M-H-	241.2175	2.20	Fatty Acyls (FA)	Fatty Acids & Conjugates	Straight/Branched

Table S2. Continued

Lipid	Formula	Ion	m/z	t_R	Lipid Category	Lipid Class	Lipid Subclass
C16:0	C16H32O2	M-H-	255.2331	2.79	Fatty Acyls (FA)	Fatty Acids & Conjugates	Straight/Branched
hydroxy C15:0	C15H30O3	M-H-	257.2122	1.38	Fatty Acyls (FA)	Fatty Acids & Conjugates	Hydroxy Fatty Acids
hydroxy C16:0	C16H32O3	M-H-	271.2279	1.63	Fatty Acyls (FA)	Fatty Acids & Conjugates	Hydroxy Fatty Acids
hydroxy C17:0	C17H34O3	M-H-	285.2435	1.84	Fatty Acyls (FA)	Fatty Acids & Conjugates	Hydroxy Fatty Acids
C16:0 amide	C16H33NO	M+H+	256.2635	1.87	Fatty Acyls (FA)	Fatty Amides	Primary amides
C18:0 amide	C18H37NO	M+H+	284.2948	2.72	Fatty Acyls (FA)	Fatty Amides	Primary amides
FL_669_8.4min	C38H72N2O7	M+H+	669.5412	8.38	Fatty Acyls (FA)	Fatty Amides	N-acyl amines
FL_669_8.7min	C38H72N2O7	M+H+	669.5412	8.67	Fatty Acyls (FA)	Fatty Amides	N-acyl amines
FL_655_7.2min	C37H70N2O7	M+H+	655.5256	7.22	Fatty Acyls (FA)	Fatty Amides	N-acyl amines
FL_655_7.5min	C37H70N2O7	M+H+	655.5256	7.50	Fatty Acyls (FA)	Fatty Amides	N-acyl amines
FL_641_6.3min	C36H68N2O7	M+H+	641.5099	6.28	Fatty Acyls (FA)	Fatty Amides	N-acyl amines
FL_641_6.5min	C36H68N2O7	M+H+	641.5099	6.53	Fatty Acyls (FA)	Fatty Amides	N-acyl amines
FL_627_5.5min	C35H66N2O7	M+H+	627.4943	5.48	Fatty Acyls (FA)	Fatty Amides	N-acyl amines
FL_627_5.7min	C35H66N2O7	M+H+	627.4943	5.68	Fatty Acyls (FA)	Fatty Amides	N-acyl amines
FL_627_5.9min	C35H66N2O7	M+H+	627.4943	5.92	Fatty Acyls (FA)	Fatty Amides	N-acyl amines
mono_FL_431	C22H42N2O6	M+H+	431.3116	1.28	Fatty Acyls (FA)	Fatty Amides	N-acyl amines
mono_FL_417	C21H40N2O6	M+H+	417.2959	1.14	Fatty Acyls (FA)	Fatty Amides	N-acyl amines
GlyL_568	C34H65NO5	M+H+	568.4935	8.44	Fatty Acyls (FA)	Fatty Amides	N-acyl amines
GlyL _554_7.3min	C33H63NO5	M+H+	554.4784	7.26	Fatty Acyls (FA)	Fatty Amides	N-acyl amines
GlyL _554_7.6min	C33H63NO5	M+H+	554.4784	7.56	Fatty Acyls (FA)	Fatty Amides	N-acyl amines
GlyL _540_6.3min	C32H61NO5	M+H+	540.4628	6.34	Fatty Acyls (FA)	Fatty Amides	N-acyl amines
GlyL _540_6.5min	C32H61NO5	M+H+	540.4628	6.53	Fatty Acyls (FA)	Fatty Amides	N-acyl amines
mono_ GlyL _330	C18H35NO4	M+H+	330.2639	1.31	Fatty Acyls (FA)	Fatty Amides	N-acyl amines
Unknown_1273	C70H134N3O14P	M+H+	1272.9676	14.51	Fatty Acyls (FA)	Fatty Amides	N-acyl amines
Unknown_1259	C69H132N3O14P	M+H+	1258.9520	14.49	Fatty Acyls (FA)	Fatty Amides	N-acyl amines
Unknown_1245	C68H130N3O14P	M+H+	1244.9363	14.42	Fatty Acyls (FA)	Fatty Amides	N-acyl amines

Table S2. Continued

Lipid	Formula	Ion	m/z	t_R	Lipid Category	Lipid Class	Lipid Subclass
Unknown_1231	C67H128N3O14P	M+H+	1230.9207	14.39	Fatty Acyls (FA)	Fatty Amides	N-acyl amines
PE (25:0)	C30H60NO8P	M+H+	594.4129	3.42	Glycerophospholipids (GP)	Glycerophosphoethanolamines (PE)	Diacyl PE
PE (26:0)_3.6min	C31H62NO8P	M+H+	608.4286	3.64	Glycerophospholipids (GP)	Glycerophosphoethanolamines (PE)	Diacyl PE
PE (26:0)_3.8min	C31H62NO8P	M+H+	608.4286	3.78	Glycerophospholipids (GP)	Glycerophosphoethanolamines (PE)	Diacyl PE
PE (26:0)_3.9min	C31H62NO8P	M+H+	608.4286	3.91	Glycerophospholipids (GP)	Glycerophosphoethanolamines (PE)	Diacyl PE
PE (26:1) *	C31H60NO8P	M+H+	606.4129	3.3	Glycerophospholipids (GP)	Glycerophosphoethanolamines (PE)	Diacyl PE
PE (27:0)_4.2min	C32H64NO8P	M+H+	622.4442	4.18	Glycerophospholipids (GP)	Glycerophosphoethanolamines (PE)	Diacyl PE
PE (27:0)_4.3min	C32H64NO8P	M+H+	622.4442	4.27	Glycerophospholipids (GP)	Glycerophosphoethanolamines (PE)	Diacyl PE
PE (27:1) *	C32H624NO8P	M+H+	620.4286	3.5	Glycerophospholipids (GP)	Glycerophosphoethanolamines (PE)	Diacyl PE
PE (28:0)_4.7min	C33H66NO8P	M+H+	636.4599	4.68	Glycerophospholipids (GP)	Glycerophosphoethanolamines (PE)	Diacyl PE
PE (28:0)_4.9min	C33H66NO8P	M+H+	636.4599	4.89	Glycerophospholipids (GP)	Glycerophosphoethanolamines (PE)	Diacyl PE
PE (28:0)_5.1min	C33H66NO8P	M+H+	636.4599	5.10	Glycerophospholipids (GP)	Glycerophosphoethanolamines (PE)	Diacyl PE
PE (28:1) *	C33H64NO8P	M+H+	634.4442	4.2	Glycerophospholipids (GP)	Glycerophosphoethanolamines (PE)	Diacyl PE
PE (29:0)_5.4min	C34H68NO8P	M+H+	650.4755	5.35	Glycerophospholipids (GP)	Glycerophosphoethanolamines (PE)	Diacyl PE
PE (29:0)_5.6min	C34H68NO8P	M+H+	650.4755	5.58	Glycerophospholipids (GP)	Glycerophosphoethanolamines (PE)	Diacyl PE
PE (29:1)*	C34H66NO8P	M+H+	648.4599	4.5	Glycerophospholipids (GP)	Glycerophosphoethanolamines (PE)	Diacyl PE
PE (30:0)_6.1min	C35H70NO8P	M+H+	664.4912	6.11	Glycerophospholipids (GP)	Glycerophosphoethanolamines (PE)	Diacyl PE
PE (30:0)_6.4min	C35H70NO8P	M+H+	664.4912	6.41	Glycerophospholipids (GP)	Glycerophosphoethanolamines (PE)	Diacyl PE
PE (30:0)_6.7min	C35H70NO8P	M+H+	664.4912	6.74	Glycerophospholipids (GP)	Glycerophosphoethanolamines (PE)	Diacyl PE
PE (30:1)*	C35H68NO8P	M+H+	662.4755	5.4	Glycerophospholipids (GP)	Glycerophosphoethanolamines (PE)	Diacyl PE
PE (31:0)_7.1min	C36H72NO8P	M+H+	678.5068	7.13	Glycerophospholipids (GP)	Glycerophosphoethanolamines (PE)	Diacyl PE

Table S2. Continued

Lipid	Formula	Ion	m/z	t_R	Lipid Category	Lipid Class	Lipid Subclass
PE (31:0)_7.4min	C36H72NO8P	M+H+	678.5068	7.40	Glycerophospholipids (GP)	Glycerophosphoethanolamines (PE)	Diacyl PE
PE (31:1)*	C36H70NO8P	M+H+	676.4912	5.9	Glycerophospholipids (GP)	Glycerophosphoethanolamines (PE)	Diacyl PE
PE (32:0)_8.2min	C37H74NO8P	M+H+	692.5229	8.18	Glycerophospholipids (GP)	Glycerophosphoethanolamines (PE)	Diacyl PE
PE (32:0)_8.5min	C37H74NO8P	M+H+	692.5229	8.54	Glycerophospholipids (GP)	Glycerophosphoethanolamines (PE)	Diacyl PE
PE (32:0)_9.0min	C37H74NO8P	M+H+	692.5229	8.96	Glycerophospholipids (GP)	Glycerophosphoethanolamines (PE)	Diacyl PE
PE (32:1)_7.0min	C37H72NO8P	M+H+	690.5072	6.99	Glycerophospholipids (GP)	Glycerophosphoethanolamines (PE)	Diacyl PE
PE (32:1)_7.2min	C37H72NO8P	M+H+	690.5072	7.22	Glycerophospholipids (GP)	Glycerophosphoethanolamines (PE)	Diacyl PE
PE (33:0)_9.9min	C38H76NO8P	M+H+	706.5386	9.86	Glycerophospholipids (GP)	Glycerophosphoethanolamines (PE)	Diacyl PE
PE (33:0)_10.0min	C38H76NO8P	M+H+	706.5386	10.03	Glycerophospholipids (GP)	Glycerophosphoethanolamines (PE)	Diacyl PE
PE (33:1)_7.6min	C38H74NO8P	M+H+	704.5229	7.62	Glycerophospholipids (GP)	Glycerophosphoethanolamines (PE)	Diacyl PE
PE (33:1)_8.1min	C38H74NO8P	M+H+	704.5229	8.05	Glycerophospholipids (GP)	Glycerophosphoethanolamines (PE)	Diacyl PE
PE (34:1)	C39H76NO8P	M+H+	718.5386	9.23	Glycerophospholipids (GP)	Glycerophosphoethanolamines (PE)	Diacyl PE
PE (P-25:0)	C30H60NO7P	M-H-	576.4035	3.78	Glycerophospholipids (GP)	Glycerophosphoethanolamines (PE)	1-(1Z-alkenyl),2-acylPE (PE P)
PE (P-26:0)_4.1min	C31H62NO7P	M-H-	590.4191	4.07	Glycerophospholipids (GP)	Glycerophosphoethanolamines (PE)	1-(1Z-alkenyl),2-acylPE (PE P)
PE (P-26:0)_4.3min	C31H62NO7P	M-H-	590.4191	4.30	Glycerophospholipids (GP)	Glycerophosphoethanolamines (PE)	1-(1Z-alkenyl),2-acylPE (PE P)
PE (P-26:0)_4.4min	C31H62NO7P	M-H-	590.4191	4.44	Glycerophospholipids (GP)	Glycerophosphoethanolamines (PE)	1-(1Z-alkenyl),2-acylPE (PE P)
PE (P-27:0)_4.7min	C32H64NO7P	M-H-	604.4348	4.66	Glycerophospholipids (GP)	Glycerophosphoethanolamines (PE)	1-(1Z-alkenyl),2-acylPE (PE P)
PE (P-27:0)_4.9min	C32H64NO7P	M-H-	604.4348	4.86	Glycerophospholipids (GP)	Glycerophosphoethanolamines (PE)	1-(1Z-alkenyl),2-acylPE (PE P)
PE (P-28:0)_5.3min	C33H66NO7P	M-H-	618.4504	5.32	Glycerophospholipids (GP)	Glycerophosphoethanolamines (PE)	1-(1Z-alkenyl),2-acylPE (PE P)
PE (P-28:0)_5.6min	C33H66NO7P	M-H-	618.4504	5.61	Glycerophospholipids (GP)	Glycerophosphoethanolamines (PE)	1-(1Z-alkenyl),2-acylPE (PE P)
PE (P-28:0)_5.8min	C33H66NO7P	M-H-	618.4504	5.82	Glycerophospholipids (GP)	Glycerophosphoethanolamines (PE)	1-(1Z-alkenyl),2-acylPE (PE P)

Table S2. Continued

Lipid	Formula	Ion	m/z	t_R	Lipid Category	Lipid Class	Lipid Subclass
PE (P-29:0)_6.2min	C34H68NO7P	M-H-	632.4661	6.17	Glycerophospholipids (GP)	Glycerophosphoethanolamines (PE)	1-(1Z-alkenyl),2-acylPE (PE P)
PE (P-29:0)_6.4min	C34H68NO7P	M-H-	632.4661	6.41	Glycerophospholipids (GP)	Glycerophosphoethanolamines (PE)	1-(1Z-alkenyl),2-acylPE (PE P)
PE (P-30:0)_7.0min	C35H70NO7P	M-H-	646.4817	7.03	Glycerophospholipids (GP)	Glycerophosphoethanolamines (PE)	1-(1Z-alkenyl),2-acylPE (PE P)
PE (P-30:0)_7.4min	C35H70NO7P	M-H-	646.4817	7.36	Glycerophospholipids (GP)	Glycerophosphoethanolamines (PE)	1-(1Z-alkenyl),2-acylPE (PE P)
PE (P-31:0)	C36H72NO7P	M-H-	660.4974	8.50	Glycerophospholipids (GP)	Glycerophosphoethanolamines (PE)	1-(1Z-alkenyl),2-acylPE (PE P)
LPE (15:0)	C20H42NO7P	M-H-	438.2627	1.14	Glycerophospholipids (GP)	Glycerophosphoethanolamines (PE)	MonoacylPE (LPE)
LPE (16:0)	C21H44NO7P	M-H-	452.2783	1.41	Glycerophospholipids (GP)	Glycerophosphoethanolamines (PE)	MonoacylPE (LPE)
LPE (17:0)	C22H46NO7P	M-H-	466.2940	1.68	Glycerophospholipids (GP)	Glycerophosphoethanolamines (PE)	MonoacylPE (LPE)
LPE (P-13:0)	C18H38NO6P	M-H-	394.2364	0.99	Glycerophospholipids (GP)	Glycerophosphoethanolamines (PE)	1Z-alkenylPE (LPE P)
LPE (P-14:0)	C19H40NO6P	M-H-	408.2521	1.18	Glycerophospholipids (GP)	Glycerophosphoethanolamines (PE)	1Z-alkenylPE (LPE P)
LPE (P-15:0)	C20H42NO6P	M-H-	422.2677	1.31	Glycerophospholipids (GP)	Glycerophosphoethanolamines (PE)	1Z-alkenylPE (LPE P)
LPE (P-16:0)	C21H44NO6P	M-H-	436.2834	1.57	Glycerophospholipids (GP)	Glycerophosphoethanolamines (PE)	1Z-alkenylPE (LPE P)
PI (27:0)	C36H69O13P	M-H-	739.4403	2.55	Glycerophospholipids (GP)	Glycerophosphoinositols (PI)	diacyl PI
PI (28:0)	C37H71O13P	M-H-	753.4560	2.82	Glycerophospholipids (GP)	Glycerophosphoinositols (PI)	diacyl PI
PI (29:0)	C38H73O13P	M-H-	767.4716	3.18	Glycerophospholipids (GP)	Glycerophosphoinositols (PI)	diacyl PI
PI (30:0)	C39H75O13P	M-H-	781.4873	3.42	Glycerophospholipids (GP)	Glycerophosphoinositols (PI)	diacyl PI
PI (31:0)	C40H77O13P	M-H-	795.5029	3.91	Glycerophospholipids (GP)	Glycerophosphoinositols (PI)	diacyl PI
PS (28:0)	C34H66NO10P	M-H-	678.4352	3.9	Glycerophospholipids (GP)	Glycerophosphoserines (PS)	diacyl PS
PS (29:0)	C35H68NO10P	M-H-	692.4508	4.6	Glycerophospholipids (GP)	Glycerophosphoserines (PS)	diacyl PS
PS (30:0)	C36H70NO10P	M-H-	706.4665	5.1	Glycerophospholipids (GP)	Glycerophosphoserines (PS)	diacyl PS
PS (31:0)	C37H72NO10P	M-H-	720.4821	5.3	Glycerophospholipids (GP)	Glycerophosphoserines (PS)	diacyl PS

Table S2. Continued

Lipid	Formula	Ion	m/z	t_R	Lipid Category	Lipid Class	Lipid Subclass
sphinganine C15	C15H33NO2	M+H+	260.2584	1.04	Sphingolipids (SP)	Sphingoid bases	Sphinganines
sphinganine C16	C16H35NO2	M+H+	274.2742	1.24	Sphingolipids (SP)	Sphingoid bases	Sphinganines
sphinganine C17	C17H37NO2	M+H+	288.2899	1.34	Sphingolipids (SP)	Sphingoid bases	Sphinganines
sphinganine C18	C18H39NO2	M+H+	302.3056	1.67	Sphingolipids (SP)	Sphingoid bases	Sphinganines
sphinganine C19	C19H41NO2	M+H+	316.3212	1.87	Sphingolipids (SP)	Sphingoid bases	Sphinganines
sphinganine C20	C20H43NO2	M+H+	330.3369	2.33	Sphingolipids (SP)	Sphingoid bases	Sphinganines
hydroxy sphinganine C18	C18H39NO3	M+H+	318.3003	1.31	Sphingolipids (SP)	Sphingoid bases	Sphinganines
hydroxy sphinganine C19	C19H41NO3	M+H+	332.3159	1.45	Sphingolipids (SP)	Sphingoid bases	Sphinganines
keto-sphinganine C15	C15H31NO2	M+H+	258.2428	1.04	Sphingolipids (SP)	Sphingoid bases	Sphinganines
keto-sphinganine C16	C16H33NO2	M+H+	272.2584	1.21	Sphingolipids (SP)	Sphingoid bases	Sphinganines
keto-sphinganine C17	C17H35NO2	M+H+	286.2741	1.31	Sphingolipids (SP)	Sphingoid bases	Sphinganines
keto-sphinganine C18	C18H37NO2	M+H+	300.2897	1.60	Sphingolipids (SP)	Sphingoid bases	Sphinganines
keto-sphinganine C19	C19H39NO2	M+H+	314.3054	1.80	Sphingolipids (SP)	Sphingoid bases	Sphinganines
keto-sphinganine C20	C20H41NO2	M+H+	328.3210	2.26	Sphingolipids (SP)	Sphingoid bases	Sphinganines
hydroxy keto-sphinganine C18	C18H37NO2	M+H+	316.2846	1.25	Sphingolipids (SP)	Sphingoid bases	Sphinganines
hydroxy keto-sphinganine C19	C19H39NO2	M+H+	330.3003	1.41	Sphingolipids (SP)	Sphingoid bases	Sphinganines
deoxy-sphinganine C17	C17H37NO	M+H+	272.2948	1.48	Sphingolipids (SP)	Sphingoid bases	Sphingoid base analogs
deoxy-sphinganine C18	C18H39NO	M+H+	286.3104	1.84	Sphingolipids (SP)	Sphingoid bases	Sphingoid base analogs
deoxy-sphinganine C19	C19H41NO	M+H+	300.3261	2.09	Sphingolipids (SP)	Sphingoid bases	Sphingoid base analogs
oxidised dihydroCer C34	C34H67NO4	M+Na	576.4968	6.80	Sphingolipids (SP)	Ceramides (Cer)	Dihydroceramides (dihydroCer)
dihydroCer C32_5.0min	C32H65NO4	M+Na	550.4815	5.02	Sphingolipids (SP)	Ceramides (Cer)	Dihydroceramides (dihydroCer)
dihydroCer C32_5.2min	C32H65NO4	M+Na	550.4815	5.19	Sphingolipids (SP)	Ceramides (Cer)	Dihydroceramides (dihydroCer)
dihydroCer C32_5.4min	C32H65NO4	M+Na	550.4815	5.39	Sphingolipids (SP)	Ceramides (Cer)	Dihydroceramides (dihydroCer)
dihydroCer C33_5.8min	C33H67NO4	M+H+	542.5146	5.77	Sphingolipids (SP)	Ceramides (Cer)	Dihydroceramides (dihydroCer)

Table S2. Continued

Lipid	Formula	Ion	m/z	t_R	Lipid Category	Lipid Class	Lipid Subclass
dihydroCer C33_6.0min	C33H67NO4	M+H+	542.5146	5.97	Sphingolipids (SP)	Ceramides (Cer)	Dihydroceramides (dihydroCer)
dihydroCer C33_6.2min	C33H67NO4	M+H+	542.5146	6.21	Sphingolipids (SP)	Ceramides (Cer)	Dihydroceramides (dihydroCer)
dihydroCer C34_6.6min	C34H69NO4	M+H+	556.5303	6.63	Sphingolipids (SP)	Ceramides (Cer)	Dihydroceramides (dihydroCer)
dihydroCer C34_6.9min	C34H69NO4	M+H+	556.5303	6.90	Sphingolipids (SP)	Ceramides (Cer)	Dihydroceramides (dihydroCer)
dihydroCer C34_7.2min	C34H69NO4	M+H+	556.5303	7.19	Sphingolipids (SP)	Ceramides (Cer)	Dihydroceramides (dihydroCer)
dihydroCer C35_7.7min	C35H71NO4	M+H+	570.5459	7.66	Sphingolipids (SP)	Ceramides (Cer)	Dihydroceramides (dihydroCer)
dihydroCer C35_8.0min	C35H71NO4	M+H+	570.5459	8.01	Sphingolipids (SP)	Ceramides (Cer)	Dihydroceramides (dihydroCer)
dihydroCer C36	C36H73NO4	M+H+	584.5616	8.84	Sphingolipids (SP)	Ceramides (Cer)	Dihydroceramides (dihydroCer)
deoxy-dihydroCer C34_7.5min	C34H69NO3	[M+Na]	562.5179	7.48	Sphingolipids (SP)	Ceramides (Cer)	Dihydroceramides (dihydroCer)
deoxy-dihydroCer C34_7.8min	C34H69NO3	[M+Na]	562.5179	7.76	Sphingolipids (SP)	Ceramides (Cer)	Dihydroceramides (dihydroCer)
deoxy-dihydroCer C35_8.7min	C35H71NO3	[M+Na]	576.5332	8.67	Sphingolipids (SP)	Ceramides (Cer)	Dihydroceramides (dihydroCer)
deoxy-dihydroCer C35_9.0min	C35H71NO3	[M+Na]	576.5332	9.03	Sphingolipids (SP)	Ceramides (Cer)	Dihydroceramides (dihydroCer)
deoxy-dihydroCer C36	C36H73NO3	[M+Na]	590.5492	9.98	Sphingolipids (SP)	Ceramides (Cer)	Dihydroceramides (dihydroCer)
Cer PE C33	C35H73N2O7P	M+H+	665.5232	4.93	Sphingolipids (SP)	Phosphosphingolipids	dihydroCer PE (Cer PE)
Cer PE C34_5.3min	C36H75N2O7P	M+H+	679.5389	5.26	Sphingolipids (SP)	Phosphosphingolipids	dihydroCer PE (Cer PE)
Cer PE C34_5.5min	C36H75N2O7P	M+H+	679.5389	5.48	Sphingolipids (SP)	Phosphosphingolipids	dihydroCer PE (Cer PE)
Cer PE C34_5.7min	C36H75N2O7P	M+H+	679.5389	5.68	Sphingolipids (SP)	Phosphosphingolipids	dihydroCer PE (Cer PE)
Cer PE C35_6.0min	C37H77N2O7P	M+H+	693.5545	6.04	Sphingolipids (SP)	Phosphosphingolipids	dihydroCer PE (Cer PE)
Cer PE C35_6.3min	C37H77N2O7P	M+H+	693.5545	6.31	Sphingolipids (SP)	Phosphosphingolipids	dihydroCer PE (Cer PE)
Cer PE C36	C38H79N2O7P	M+H+	707.5702	6.99	Sphingolipids (SP)	Phosphosphingolipids	dihydroCer PE (Cer PE)
Cer PI C33	C39H78NO12P	M+H+	784.5334	2.93	Sphingolipids (SP)	Phosphosphingolipids	dihydroCer PI (Cer PI)

Table S2. Continued

Lipid	Formula	Ion	m/z	t_R	Lipid Category	Lipid Class	Lipid Subclass
Cer PI C34	C40H80NO12P	M+H+	798.5495	3.11	Sphingolipids (SP)	Phosphosphingolipids	dihydroCer PI (Cer PI)
Cer PI C35	C41H82NO12P	M+H+	812.5652	3.57	Sphingolipids (SP)	Phosphosphingolipids	dihydroCer PI (Cer PI)
Cer PI C36	C42H84NO12P	M+H+	826.5809	3.84	Sphingolipids (SP)	Phosphosphingolipids	dihydroCer PI (Cer PI)
α-Gal Cer C33	C39H77NO9	M+H+	704.5671	4.99	Sphingolipids (SP)	Neutral glycosphingolipids	Simple Glc series
α-Gal Cer C34_5.5min	C40H79NO9	M+H+	718.5828	5.51	Sphingolipids (SP)	Neutral glycosphingolipids	Simple Glc series
α-Gal Cer C34_5.7min	C40H79NO9	M+H+	718.5828	5.70	Sphingolipids (SP)	Neutral glycosphingolipids	Simple Glc series
α-Gal Cer C35_6.3min	C41H81NO9	M+H+	732.5984	6.34	Sphingolipids (SP)	Neutral glycosphingolipids	Simple Glc series
α-Gal Cer C35_6.6min	C41H81NO9	M+H+	732.5984	6.60	Sphingolipids (SP)	Neutral glycosphingolipids	Simple Glc series
α-Gal Cer C36	C42H83NO9	M+H+	746.6141	7.33	Sphingolipids (SP)	Neutral glycosphingolipids	Simple Glc series
16:0 SM	C39H79N2O6P	M+HCOO-	747.5658	6.17	Sphingolipids (SP)	Internal Standard	Internal Standard
16:0 SM	C39H79N2O6P	M+H+	703.5749	6.17	Sphingolipids (SP)	Internal Standard	Internal Standard

DG: diacylglycerols; C: carbon; FL: flavolipin; mono: monoacyl; GlyL: glycine lipid; Gal: galactosyl; SM: sphingomyelin

* May contain more than one peak/isomer

Where possible LipidMaps classification & nomenclature is used throughout.

GP abbreviation is based on sum composition which accounts for the total number of carbons and double bonds in the fatty acyl chains (number of carbons: number double bonds). For example, PE (30:0) denotes a diacyl PE whereby the sum of carbons in the 2 acyl chains is 30 and both acyl chains are saturated (no double bond); possibilities include a C17:0 and C13:0; C15:0 and C15:0; C14:0 and C16:0 and so on. Fatty acyl chains may also be branched/unbranched. Where there are more than one chromatographic peak GP are also distinguished on their elution time (min), for example PE (30:0)_6.1 min, and indicates the possibilities of numerous combinations of fatty acyls (chain lengths and branching)

Bacteroides SP abbreviation is based on the combined total number of carbons on the sphinganine backbone and the fatty acyl chain. Where there is more than one peak, they are also distinguished based on their elution or retention time (t_R in min). For example, Cer PE C34_5.3min has a combined total of 34 carbons in the sphinganine backbone and fatty acyl chain and elutes at 5.3 min. Like GP there are numerous combinations of sphinganine and fatty acyl chain length as well as branching and non-branching on the sphinganine and/or the fatty acyl component. There is evidence to suggest that debranched SP elute first with subsequent elution of mono-branched and unbranched isomers (Oh et al. 2021).
